# Image analysis reveals molecularly distinct patterns of TILs in NSCLC associated with treatment outcome

**DOI:** 10.1038/s41698-022-00277-5

**Published:** 2022-06-03

**Authors:** Ruiwen Ding, Prateek Prasanna, Germán Corredor, Cristian Barrera, Philipp Zens, Cheng Lu, Priya Velu, Patrick Leo, Niha Beig, Haojia Li, Paula Toro, Sabina Berezowska, Vipul Baxi, David Balli, Merzu Belete, David L. Rimm, Vamsidhar Velcheti, Kurt Schalper, Anant Madabhushi

**Affiliations:** 1 Case Western Reserve University, Cleveland, OH USA; 2 Stony Brook University, New York, NY USA; 3 Louis Stokes Cleveland VA Medical Center, Cleveland, OH USA; 4 Institute of Pathology, University of Bern, Bern, Switzerland; 5 Graduate School for Health Sciences, University of Bern, Bern, Switzerland; 6 Weill Cornell Medical College, New York, NY USA; 7 Department of Laboratory Medicine and Pathology, Institute of Pathology, Lausanne University Hospital and University of Lausanne, Lausanne, Switzerland; 8 Bristol Myers Squibb, New York, NY USA; 9 Yale University, New Haven, CT USA; 10 New York University, New York, NY USA

**Keywords:** Cancer, Non-small-cell lung cancer

## Abstract

Despite known histological, biological, and clinical differences between lung adenocarcinoma (LUAD) and squamous cell carcinoma (LUSC), relatively little is known about the spatial differences in their corresponding immune contextures. Our study of over 1000 LUAD and LUSC tumors revealed that computationally derived patterns of tumor-infiltrating lymphocytes (TILs) on H&E images were different between LUAD (*N* = 421) and LUSC (*N* = 438), with TIL density being prognostic of overall survival in LUAD and spatial arrangement being more prognostically relevant in LUSC. In addition, the LUAD-specific TIL signature was associated with OS in an external validation set of 100 NSCLC treated with more than six different neoadjuvant chemotherapy regimens, and predictive of response to therapy in the clinical trial CA209-057 (*n* = 303). In LUAD, the prognostic TIL signature was primarily comprised of CD4^+^ T and CD8^+^ T cells, whereas in LUSC, the immune patterns were comprised of CD4^+^ T, CD8^+^ T, and CD20^+^ B cells. In both subtypes, prognostic TIL features were associated with transcriptomics-derived immune scores and biological pathways implicated in immune recognition, response, and evasion. Our results suggest the need for histologic subtype-specific TIL-based models for stratifying survival risk and predicting response to therapy. Our findings suggest that predictive models for response to therapy will need to account for the unique morphologic and molecular immune patterns as a function of histologic subtype of NSCLC.

## Introduction

Lung cancer results in more than one million deaths worldwide each year^1^. Non-small cell lung cancers (NSCLC) comprise around 85% of all lung malignancies in the United States^2^ and have an average 5-year survival rate of less than 21%^3^. The most common types of NSCLC include adenocarcinoma (LUAD) and squamous cell carcinoma (LUSC). LUAD originates from specialized glandular-like epithelial cells, and LUSC arises from flat epidermoid cells lining the lung airways. These histologic NSCLC variants differ in their pathophysiology, clinical features, prognosis, and treatment sensitivity. For example, LUAD has significantly better stage-specific 5-year overall survival (OS) as compared to LUSC^4^. In chemotherapy, a multiple-enzyme inhibitor, pemetrexed, is utilized in LUAD instead of in LUSC^5^. Further, immune subtyping using gene expression patterning shows that LUAD and LUSC have different immune checkpoint expression and microenvironment factors^6^.

Cells in the microenvironment surrounding the tumor such as stromal and immune cells are intrinsically related to tumor development, growth, and progression^7–9^. Recent findings suggest that tumor-infiltrating lymphocytes (TILs) have strong prognostic influence and can differentiate outcomes within each tumor, node, and metastasis stage (TNM). High levels of tumor-infiltrating stromal CD3^+^ TILs have significant prognostic impact on TNM staging in NSCLC across stages^7^. A number of studies have shown that TIL density has been associated with disease prognosis for several cancer types^10–16^. Some studies have suggested that the spatial patterns of TIL arrangement might potentially be even more prognostically important as compared to TIL density or count^13,17,18^. Despite known histological and pathophysiological differences between LUAD and LUSC, relatively little is known about the morphologic differences in the spatial patterns of immune cells from H&E images across stages. For example, Corredor et al. previously found that the spatial arrangement of TILs was associated with the likelihood of recurrence in early-stage NSCLC^13^ but did not consider potential differences between LUAD and LUSC. Studies have reported the importance of immune composition in determining therapeutic efficacy in various cancer types^19^. Objective measurement of molecular composition of TILs using multiplexed quantitative immunofluorescence images (QIF) showed that increased levels of CD8^+^ T cells are associated with better outcome in NSCLC^9^. However, the difference in molecular composition of prognostic TIL signatures from hematoxylin and eosin (H&E) images in LUAD and LUSC has not been rigorously studied. Understanding both morphologic and molecular differences in the immune contexture in LUAD and LUSC will help evaluate these histologic subtypes toward developing more personalized risk stratification models. Additionally, this could help to develop a more accurate prediction of benefit or response to therapies, such as chemotherapies or immune checkpoint inhibitors blockade, for LUAD and LUSC separately^20^.

**Fig. 1:**
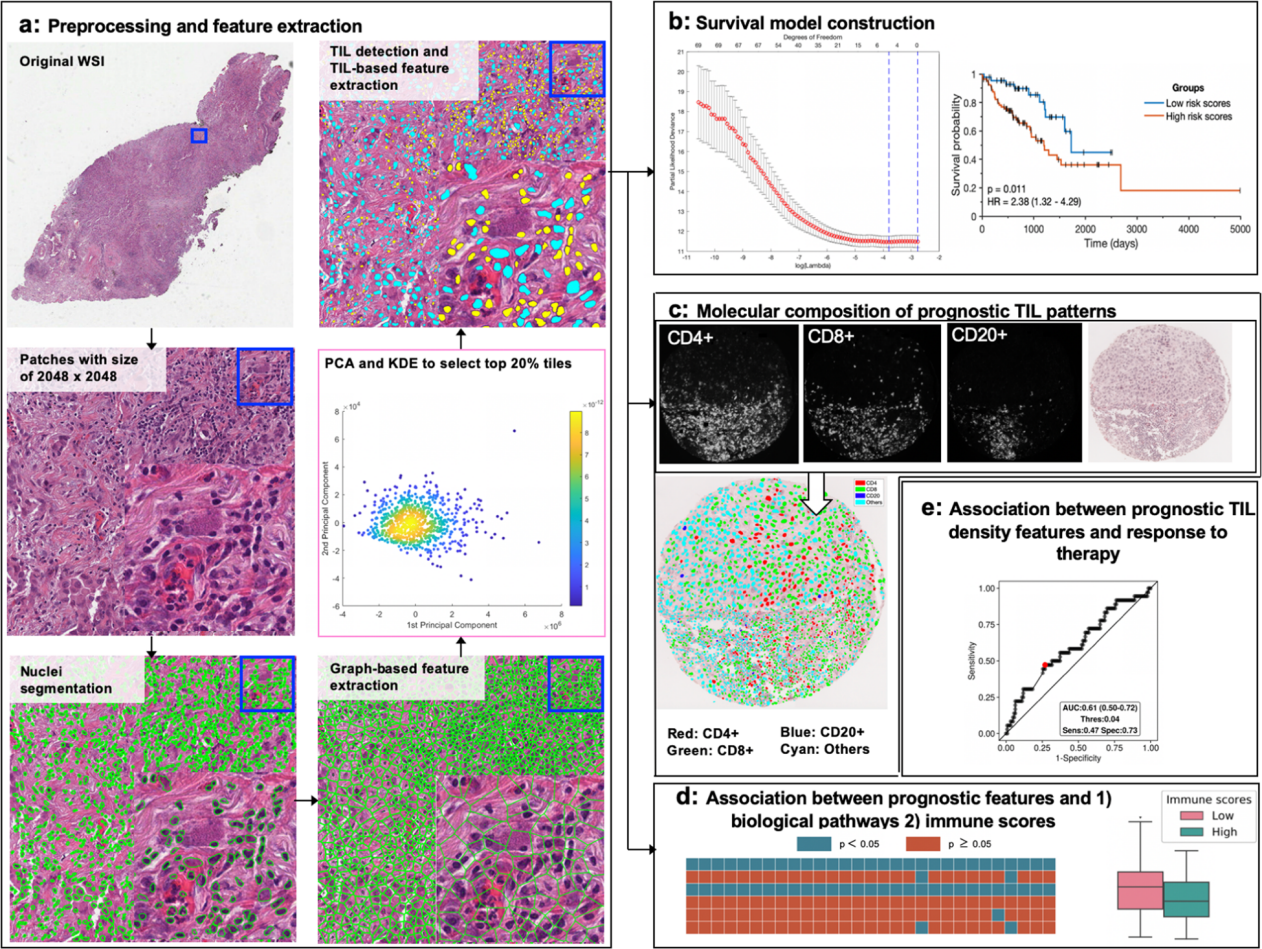
The overall procedure of this study. **a** *Preprocessing and feature extraction*. The original whole slide image of each patient was tiled into image patches of size 2048 by 2048 pixels. This was followed by automated nuclei detection using a watershed-based algorithm and graph- and shape-based feature extraction from the detected nuclei. Targeted tile selection was then performed to select the most representative tiles determined by a dimensionality reduction-based algorithm from each case. TILs were then detected using a Support Vector Machine model followed by extraction of multiple TIL-based features. **b** *Construction of prognostic models using TIL-based features* extracted from H&E images using Cox proportional hazards regression model with the least shrinkage and selection operator (LASSO) method as a feature selection tool. **c** *Determination of the molecular composition of prognostic signals* identified from H&E images by first co-registering the QIF images and their corresponding tissue microarrays (TMA) and then interrogating the molecular composition of the TIL patterns identified as prognostic from H&E images. **d** *Features-Pathway association*. The association between prognostic features from H&E images and (1) biological pathways implicated in immune recognition, response, and evasion, and (2) ISs was studied. **e** Prediction of response status using TIL density feature-predicted risk categories identified from **b** in advanced NSCLC patients treated with chemotherapy.

In this work, we use machine learning to model the morphologic and molecular differences in immune patterns in digitized H&E images and associate these patterns with OS in LUAD and LUSC across different TNM stages. We consider TIL as any lymphocyte located within tumoral tissue, which includes both intratumoral and stromal lymphocytes^21^. Specifically, we (1) investigate the spatial morphologic patterns of TILs from H&E images that are associated with OS in 421 LUAD and 438 LUSC separately using Cox proportional hazard regression models; (2) assess whether these prognostic TIL signatures for LUAD and LUSC respectively are molecularly distinct in their immune composition using QIF images for major TIL subsets (*N* = 62 for LUAD, 21 for LUSC); (3) characterize the association between the spatial patterns of TILs on H&E images with transcriptomics-derived immune scores (ISs) and biological pathways implicated in immune recognition, response, and evasion by performing single-sample gene-set enrichment analysis (ssGSEA); and (4) evaluate whether the computationally derived TIL signatures were associated with OS in 100 NSCLC patients treated with more than six different neoadjuvant chemotherapy regimens and whether the TIL signatures are predictive of response to Nivolumab and Docetaxel in 303 LUAD patients. To investigate the association between the TIL signatures and clinical outcome, we build Cox proportional hazard models separately for LUAD and LUSC using different sets of computer-extracted features such as TIL-density-based features and TIL-spatial-arrangement-based features from the H&E images. Figure 1 shows the overall methodology comprising feature extraction, region selection, risk model construction, determining molecular composition of prognostic TIL signatures, and IS and pathway association.

## Results

### Datasets and image features

Six datasets were used in this retrospective study. The first two datasets include pre-treatment whole slide images (WSIs) of H&E pathology scans of 585 LUAD patients (D_1_) and 504 LUSC patients (D_2_), at 40×, which were obtained from The Cancer Genome Atlas of National Cancer Institute (TCGA)^22,23^. The third and fourth datasets consist of pre-treatment formalin-fixed paraffin-embedded tumor sections from retrospectively sampled 126 LUAD (D_3_) and 36 LUSC (D_4_) patients collected at Yale Pathology^9^. The dataset was represented in tissue microarrays (TMA) with 0.6-mm cores from each of the paraffin blocks. The TMAs were obtained via the standard TMA preparation protocol^24^. We also acquired corresponding QIF images for D_3_ and D_4_. The general multiplexing TILs immunofluorescence staining protocol was used to simultaneously detect cytokeratin positive tumor cells and major TIL subtypes, namely CD4^+^ T, CD8^+^ T, and CD20^+^ B cells^12^. Specifically, the TMAs were subjected to antigen retrieval using EDTA buffer (Sigma-Aldrich, St Louis, MO) with pH = 8.0. With isotype-specific primary antibodies, staining for pancytokeratin, CD3, CD8, and CD20 was performed to detect epithelial tumor cells, T lymphocytes, cytotoxic T cells, and B lymphocytes. Nuclei from all cells was stained using 4′,6-diamidino-2-phenylindole. This process yielded the QIF images for each patient in D_3_ and D_4_ with each patient having one QIF image for each TIL subtype, CD4^+^ T, CD8^+^ T, and CD20^+^ B cells. The fifth dataset (D_5_) included 123 patients with locally-advanced LUAD from University of Bern in Switzerland (UBern), which were scanned using a Pannoramic P250 Flash III scanner at 20× (0.242 microns per pixel)^25^. A subset of 63 patients was treated with chemotherapy prior to resection of which 57 patients in neoadjuvant intention and 60 patients were primary resected LUAD with pathologically confirmed infiltration of lymph nodes of at least the mediastinal level (indication of a locally-advanced stage). Patients of the neoadjuvant subset received platinum-based chemotherapy in different combinations: (1) Cisplatin plus Docetaxel, (2) Carboplatin plus Paclitaxel, (3) Cisplatin plus Pemetrexed, (4) Cisplatin plus Gemcitabine, (5) Cisplatin plus Vinorelbine, (6) Cisplatin plus Etoposide, and (7) other. The sixth dataset consists of 303 patients from CA209-057^26^ (ClinicalTrials.gov number NCT01673867). Briefly, CA209-057 is a phase 3 randomized clinical trial designed to compare OS of advanced non-squamous NSCLC subjects treated with either Nivolumab (*n* = 162) or Docetaxel (*n* = 141) after failure of previous platinum-based chemotherapy. The response status (responder/non-responder) was defined by the Response Evaluation Criteria in Solid Tumors version 1.1^26^. Quality check and preprocessing (see Methods for details) resulted in six datasets for this study, including 421 TCGA-LUAD (D_1_), 438 TCGA-LUSC (D_2_), 62 Yale-LUAD (D_3_), 21 Yale-LUSC (D_4_), 100 UBern-LUAD (D_5_), and 303 CA209-057 (D_6_) cases suitable for the downstream analysis. The detailed clinicopathologic characteristics for each dataset are shown in Table 1. Corresponding clinicopathologic and outcome information from patients were obtained from the institutions at which the datasets were collected (University of Bern, Yale University, Bristol Myers Squibb) after obtaining the respective institutional review board approvals.

**Table 1:** A summary of the clinicopathologic variables in all cohorts.

		TCGA-LUAD (D_1_)		TCGA-LUSC (D_2_)		Yale-LUAD (D_3_)	Yale-LUSC (D_4_)	UBern-LUAD (D_5_)	CA209-057-LUAD (D_6_^Nivolumab^)	CA209-057-LUAD (D_6_^Docetaxel^)
Characteristics	Subgroups or values	Training *n* (%)	Testing *n* (%)	Training *n* (%)	Testing *n* (%)	CV *n* (%)	CV *n* (%)	External validation *n* (%)	External validation *n* (%)	External validation *n* (%)
Gender	Male	133 (45.2)	53 (41.7)	225 (73.5)	104 (78.8)	27 (43.5)	15 (71.4)	50 (50.0)	87 (54.0)	77 (55.0)
	Female	161 (54.8)	74 (58.3)	81 (26.5)	28 (21.2)	35 (56.5)	6 (28.6)	50 (50.0)	75 (46.0)	64 (45.0)
Tumor stage	Stage I	157 (53.4)	70 (55.1)	137 (44.8)	73 (55.3)	37 (59.6)	14(66.7)	9 (9.0)	0 (0)	0 (0)
	Stage II	80 (27.2)	28 (22.0)	106 (34.6)	36 (27.3)	5 (8.1)	7 (33.3)	11 (11.0)	0 (0)	0 (0)
	Stage III	40 (13.6)	18 (14.2)	55 (18.0)	21 (15.9)	16 (25.8)	0 (0)	70 (70.0)	10 (6.2)	13 (9.2)
	Stage IV	13 (4.4)	9 (7.1)	5 (1.6)	1 (0.8)	4 (6.5)	0 (0)	10 (10.0)	152 (93.8)	128 (90.8)
	Unknown	4 (1.4)	2 (1.6)	3 (1.0)	1 (1.8)	N/A	N/A	N/A	0 (0)	0 (0)
Mean age	years	65 ± 10.1	66 ± 9.7	67 ± 8.8	68 ± 7.6	64 ± 10.5	64 ± 7.2	62 ± 9.7	60 ± 8.9	62 ± 8.9
Mean overall survival time	days	948 ± 971	864 ± 708	992 ± 984	931 ± 924	1084 ± 854	2085 ± 958	911 ± 644	635 ± 656	437 ± 451

Using the algorithm of Veta et al.^27^, individual nuclei were then identified from tiled H&E images of size 2048 by 2048 pixels in D_1_, D_2_, D_5_, and D_6_, and from TMA spots in D_3_ and D_4_. TILs were distinguished from non-TILs in D_1_, D_2_, D_5_, and D_6_ via a support vector machine classifier^13^ (see Methods for details). After targeted tile selection for reducing computational complexity, a total of five groups of features were extracted for the downstream analysis: graph- and shape-based features on all nuclei without distinguishing TILs and non-TILs (960 features), on TILs (960 features), and on non-TILs (960 features), TIL spatial arrangement features (SpaTIL, 1400 features), and TIL density features (DenTIL, 76 features) (see Methods for details).

### Experiment 1: Spatial morphological patterns of TILs in H&E images are different between LUAD and LUSC

**Fig. 2:**
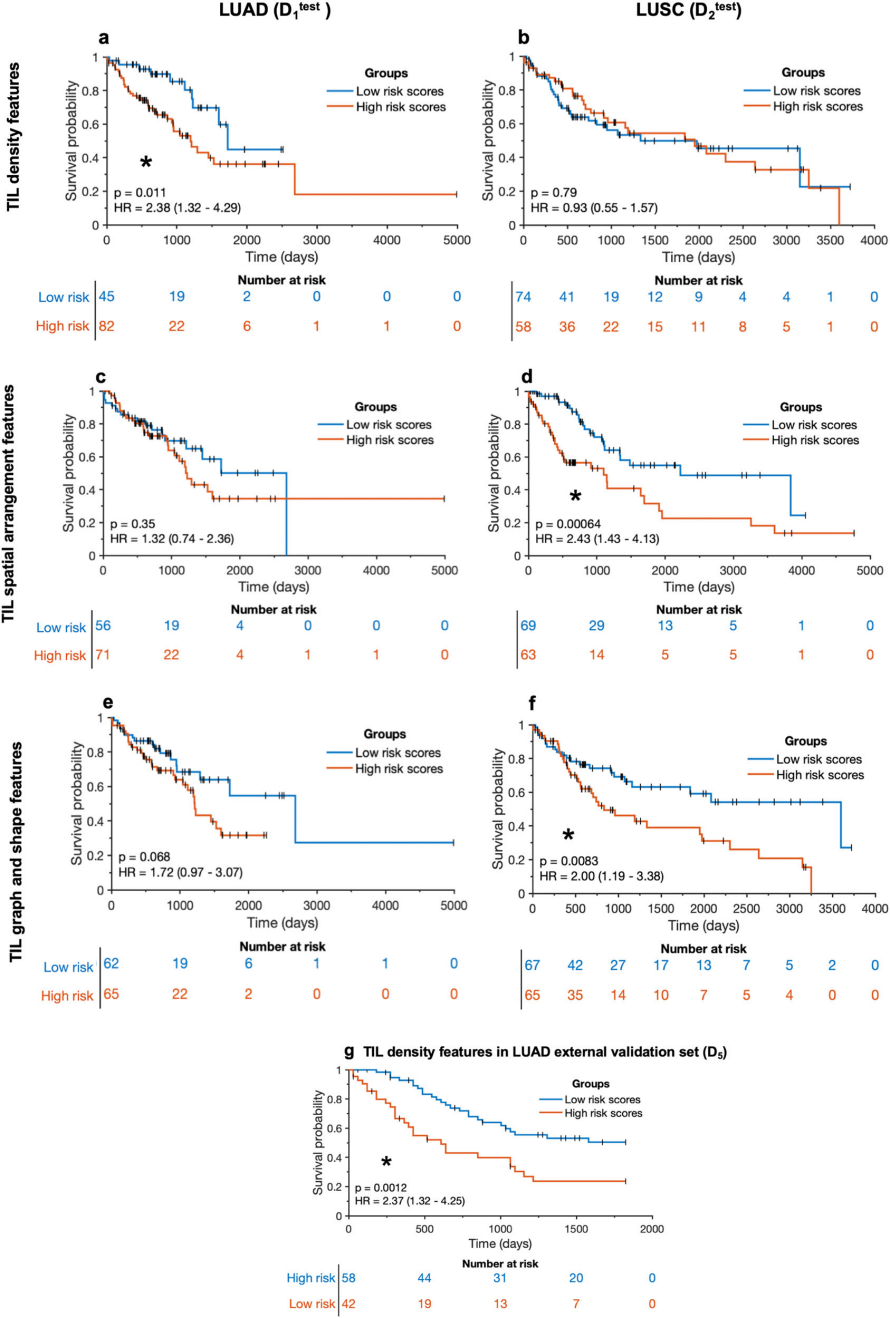
The Kaplan-Meier survival curves generated from the results of Cox model on the independent test sets of D_1_ and D_2_, namely D_1_^test^ and D_2_^test^, using M_H&E_^LUAD^ and M_H&E_^LUSC^, respectively, and external validation set D_5_. **a**, **c**, **e** represent the KM curves of LUAD cases (D_1_^test^) and **b**, **d**, **f** show the ones for LUSC cases (D_2_^test^). **a**, **b** represent the TIL density measures, **c**, **d** represent the spatial arrangement features of TILs (SpaTIL features), and **e**, **f** represent the graph- and shape-based features on TILs. **g** represents TIL density features from D_5_. Statistically significant results are highlighted with asterisks.

**Fig. 3:**
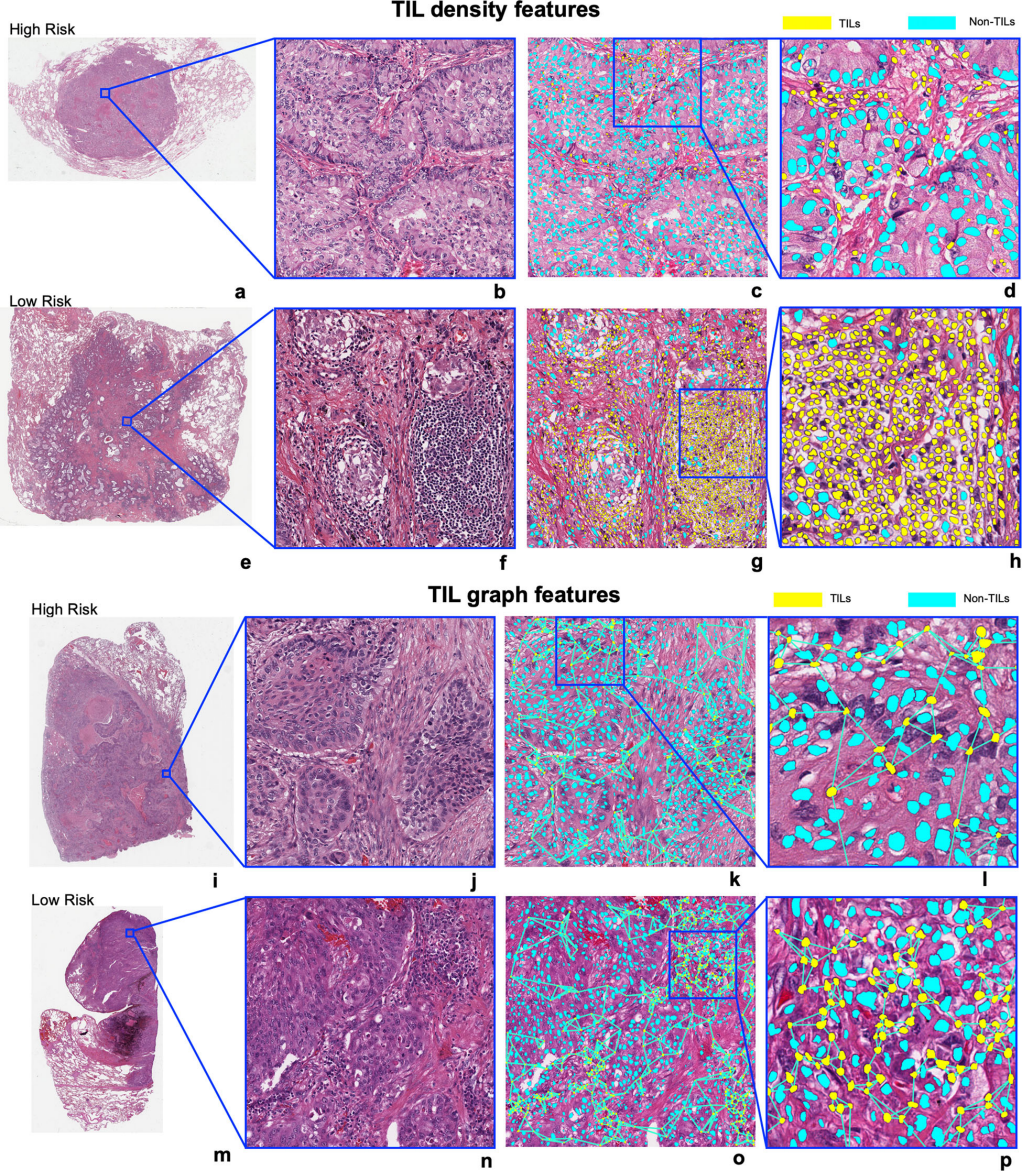
Visualization of prognostic TIL features in TCGA-LUAD and TCGA-LUSC. **a**–**h** represent the TIL density measures in LUAD. **a**, **e** show the original WSIs from a high-risk and a low-risk case, respectively. **b**, **f** show example tiles of size 2048 by 2048 pixels from them. **c**, **g** show the corresponding TIL maps highlighting the TILs in yellow and non-TILs in blue. TIL density in the low-risk patient is prominently higher than in the high-risk patient. **i**–**p** represent the visualization of Euclidean distance to three nearest neighbors of each TIL in LUSC and the distance was measured from the centroid of each TIL. **i**, **m** show the original 40× WSIs from a high-risk and a low-risk case from D_2_^test^ using M_H&E_^LUSC^, respectively. **j**, **n** show example tiles of size 2048 by 2048 pixels from them. **k**, **o** show the corresponding TIL maps highlighting the TILs in yellow and non-TILs in blue. The TIL distribution is sparser in a high-risk patient as compared to a low-risk patient.

Figure 2 shows the Kaplan-Meier survival curves obtained by applying the trained models (M_H&E_^LUAD^ and M_H&E_^LUSC^) using the training sets (D_1_^train^, *n* = 294) and (D_2_^train^, *n* = 306) with TIL-based features on the independent test sets using D_1_^test^ (*n* = 127) and D_2_^test^ (*n* = 132). In D_1_, as shown in Fig. 2a, the model (M_H&E_^LUAD^) trained using TIL density measures was statistically significantly prognostic of OS when applied to the testing set D_1_^test^ with a hazard ratio (HR) of 2.38 (95% confidence interval (CI) = 1.32–4.29, *p* value = 0.011, concordance-index (C-index) = 0.606 (95% CI 0.516–0.696, standard error (SE) = 0.046)). The TIL density signatures were also prognostic of OS in the external validation set D_5_ with an HR of 2.37 (95% CI 1.32–4.25), a *p* value of 0.0012, and a C-index of 0.659 (95% CI 0.585–0.733 S, SE = 0.038). A qualitative evaluation of TIL density features is shown in Fig. 3a–h. As may be observed, TIL density is higher in low-risk than in high-risk patients.

In D_2_, the prognostic features in the independent test set D_2_^test^ using M_H&E_^LUSC^ were broadly related to spatial distribution of TILs. Specifically, both models using (1) graph- and shape-based features on TILs shown in Fig. 2f, and (2) spatial arrangement/co-localization features on TILs and non-TILs shown in Fig. 2d were associated with OS, respectively (in D_2_^test^ of graph- and shape-based features, HR = 2.00, 95% CI = 1.19-3.38, *p* value = 0.0083, C-index = 0.561 (95% CI 0.477–0.645, SE = 0.043); in D_2_^test^ of spatial arrangement features, HR = 2.43, 95% CI = 1.43–4.13, *p* value = 0.00064, C-index = 0.649 (95% CI 0.563–0.735, SE = 0.044). A qualitative evaluation of prognostic graph- and shape-based features, specifically the distance to three nearest neighbors for each TIL, is shown in Fig. 3i–p. As may be observed, the average distance from each TIL to its three nearest neighbors is shorter in the low-risk compared to the high-risk patient. Supplementary Table 1 shows a complete list of the feature description for the prognostic features and their associated weights from the LASSO method^28^.

For both LUAD and LUSC (D_1_ and D_2_), models (M_H&E_^LUAD^ and M_H&E_^LUSC^) trained with graph- and shape-based features on all nuclei, graph- and shape-based features on non-TILs, and clinical features (gender, age, tumor stage, and cigarettes per day) did not yield statistically significant results on the independent test sets D_1_^test^ and D_2_^test^ (see Supplementary Fig. 2).

In this paper, the term “spatial distribution” generally refers to any features that involve spatial organization of cells, and this includes spatial arrangement/co-localization features and graph-based features.

### Experiment 2: Molecular composition of prognostic TIL signatures is different between LUAD and LUSC

Since TIL density measures features were found to be prognostic of OS in D_1_ (LUAD, *N* = 421) and D_5_ (LUAD, *N* = 100), this feature group was used to construct survival model (M_QIF_^LUAD^) using TIL subtypes from D_3_ (LUAD, *N* = 62). As shown in Table 2, results in D_3_ reveal that density measures of CD4^+^ T and CD8^+^ T cells were prognostic of OS in D_3_. Since both TIL spatial arrangement features and graph- and shape-based features were found to be prognostic of OS in D_2_ (LUSC, *N* = 438), these two feature groups were then used to construct survival model (M_QIF_^LUSC^) using TIL subtypes from D_4_ (LUSC, *N* = 21). Results in D_4_ suggest that the spatial interaction between CD4^+^ T and CD8^+^ T cells and the interaction between CD4^+^ T and CD20^+^ B cells were prognostic of OS in D_4_. Graph- and shape-based features extracted from both CD8^+^ T cells and the combination of CD4^+^ T cells and CD20^+^ B cells were prognostic of OS in D_4_. A complete list of cross-validated Kaplan-Meier survival curves on all different feature groups using different TIL subtypes can be found in Supplementary Figs. 3, 4 shows the visualization of prognostic features on different TIL subtypes in D_3_ and D_4_.

**Table 2:** Summary of prognostic results from the cross-validated Kaplan Meier estimation with different TIL subtypes and feature types on the QIF datasets D_3_ and D_4_.

Dataset	Feature type	TIL subtype (s)	Cross-validated results
LUAD (D_3_) *N* = 62	Density measures	CD4	HR = 2.22 (1.11–4.45), *p* = 0.024, permutation significance (PS) = 0.02, C-index = 0.638 (95% CI 0.524–0.752, SE = 0.058)
		CD8	HR = 2.16 (1.08–4.31), *p* = 0.035, PS = 0.03, C-index = 0.618 (95% CI 0.442–0.794, SE = 0.09)
LUSC (D_4_) *N* = 21	Spatial interaction	CD4 versus CD8	HR = 4.09 (1.23–13.63), *p* = 0.01, PS = 0.14, C-index = 0.646 (95% CI 0.464–0.828, SE = 0.093)
		CD4 versus CD20	HR = 3.24 (1.01–10.40), *p* = 0.038, PS = 0.06, C-index = 0.637 (95% CI 0.433–0.841, SE = 0.104)
	Graph- and shape-based features	CD8	HR = 3.80 (1.16–12.50), *p* = 0.017, PS = 0.09, C-index = 0.561 (95% CI 0.42–0.702, SE = 0.072)
		CD4 plus CD20	HR = 3.43 (1.06–11.10), *p* = 0.029, PS = 0.10, C-index = 0.567 (95% CI 0.351–0.783, SE = 0.11)

The spatial arrangement between all of the TILs and tumor cells was prognostic of OS in D_4_ (HR = 4.18 (1.19–16.90), *p* = 0.003, PS = 0.02, C-index = 0.579 (SE = 0.109))_._ Neither density measures on all TILs in D_3_ nor graph- and shape-based features on all TILs in D_4_ were statistically significantly prognostic of OS.

### Experiment 3: Prognostic TIL features are associated with ISs and biological pathways implicated in immune recognition, response, and evasion

**Fig. 4:**
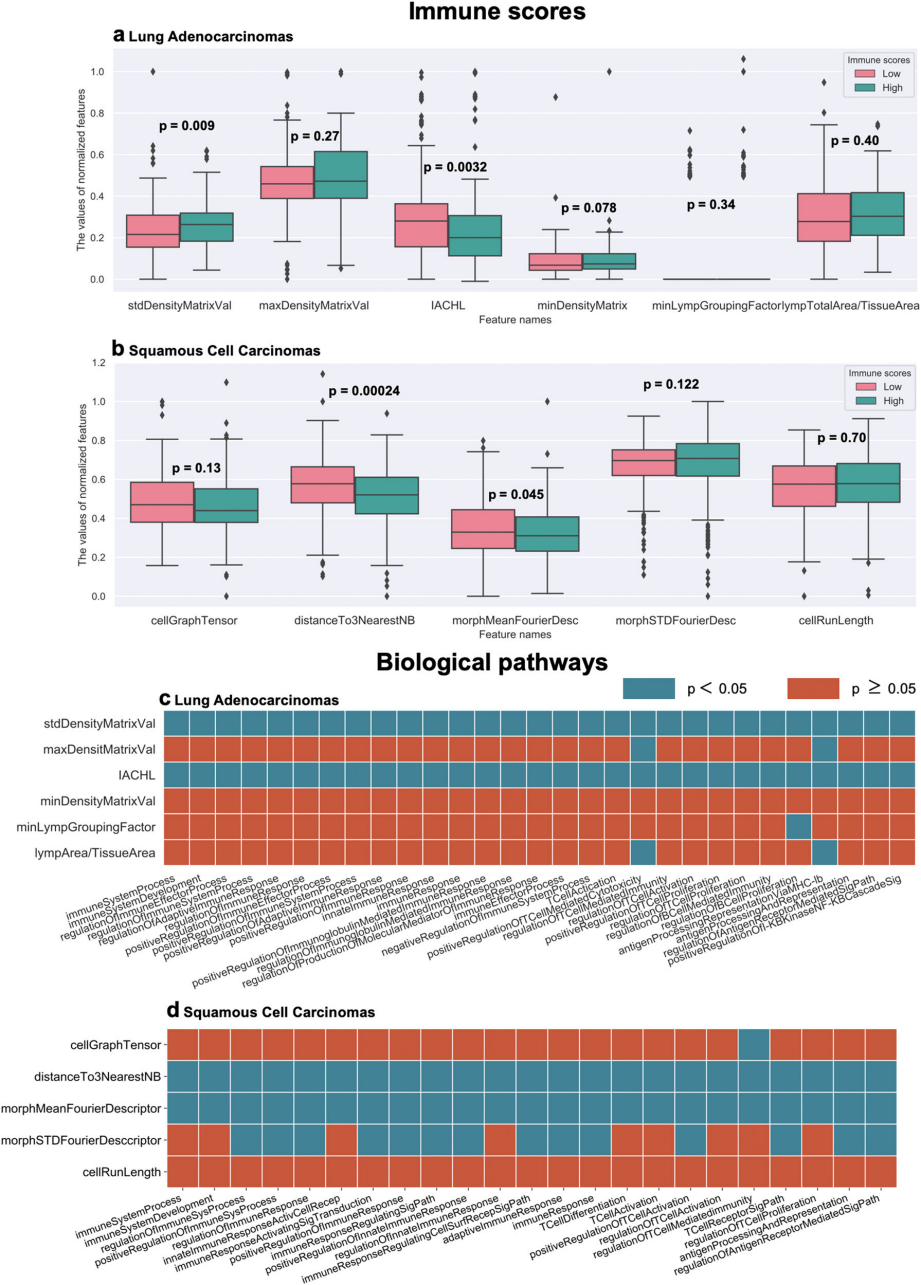
Association between prognostic features and (1) immune scores (2) biological pathways. **a**, **b** represent the distribution of the prognostic features for the low and high ISs groups. **a** is for TCGA-LUAD (D_1_) and **b** is for TCGA-LUSC (D_2_). **c**, **d** show matrices containing the statistical significance (*p* values) of the difference in the distribution of enrichment score of biological pathways in patients who had low or high feature value. The columns represent the enrichment scores and the rows represent the values of the most prognostic features. In both **c**, **d**, the statistical significance was computed using Wilcoxon Rank Sum Test.

ISs of D_1_ and D_2_ from the ESTIMATE algorithm were obtained from the publicly available website^29^. Each patient in the cohort has one corresponding IS. For LUAD cases (D_1_), the ISs range from −1355.85 to 3286.67, and for LUSC cases, they range from −1651.61 to 3198.31. Two out of the six most discriminant TIL density features were statistically significantly correlated with the ISs. These two features are the standard deviation of Density Matrix Value (DMV, the count of TILs in each grid when dividing the image tile into a 5 by 5 grid) and the range of the intersection area between the lymphocytes convex hull and the non-lymphocytes convex hull (IACHL). Median IS was used to divide the patients into those with low and high ISs. As shown in Fig. 4a, the two features significantly associated with ISs also exhibit significantly different feature values in low-high IS groups.

For LUSC (D_2_), two out of the five most discriminant graph- and shape-based features were statistically significantly associated with ISs (see Fig. 4b). They were the average of distance to three nearest neighbors of TILs (on individual TILs) and the standard deviation of Fourier shape descriptor feature of TILs (Fourier transform of the TIL contour^30^). Similar to what is shown for LUAD, in LUSC cases, the two features significantly associated with ISs also exhibit differential expression in low-high IS groups. In addition, no significant correlation was found between prognostic spatial arrangement features and ISs for LUSC.

RNA-sequencing of 20,531 genes were obtained from TCGA and they were used to investigate the biological pathways associated with the prognostic features from H&E images in D_1_ and D_2_. In LUAD (D_1_), 984 genes were significantly associated with the risk scores derived using prognostic TIL density measures. Using these genes, a total of 249 biological pathways were obtained from the Gene Ontology (GO) analysis platform^31,32^, and 29 of them were related to immune recognition, response, and evasion. Note that since this study is mainly concerned with computationally-derived immune patterns and their biological basis of them, only biological pathways related to immune activities were used to associate with prognostic features. Pathways not related to immune activities were not analyzed. In LUSC (D_2_), 155 genes were significantly associated with the risk scores derived from prognostic graph- and shape-based features of TILs. A total of 90 biological pathways were obtained from the GO platform using risk scores derived from prognostic graph- and shape-based features of TILs, and 23 of which were the most relevant to our study. No biological pathways were found using the risk scores derived from prognostic spatial arrangement features of TILs. For each of these immune-related pathways in both LUAD and LUSC, an enrichment score was assigned to each patient. The statistical significance of the difference in the distribution of ssGSEA enrichment scores and (1) the six most prognostic TIL density features (Fig. 4c) and (2) the five most prognostic graph- and shape-based features (Fig. 4d) was then calculated. As shown in Fig. 4c, for two out of six prognostic TIL density features, standard deviation of DMV and IACHL in LUAD, all of the 29 biological pathways were significantly differentially expressed in the low-high feature value groups. Similarly, as shown in Fig. 4d, for two out of five prognostic graph- and shape-based features in LUSC, distance to three nearest neighbors and mean of Fourier descriptor of the TIL contour, all of the 23 biological pathways were significantly differentially expressed in the low-high feature value groups.

### Experiment 4: Association between prognostic TIL density features and response to therapy

**Fig. 5:**
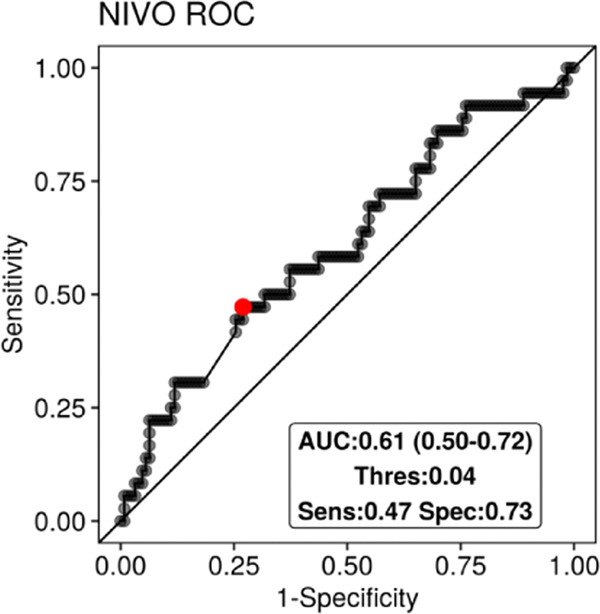
Results from Experiment 4. Receiver Operating Characteristics curves from using DenTIL-predicted risk categories to predict the response status of patients in D_6_, specifically for the 162 patients treated with Nivolumab.

The DenTIL-predicted risk categories (low or high risk) were used to predict whether the patients in D_6_ will respond to therapy or not. As shown in Fig. 5, the areas under the Receiver Operating Characteristics curve (AUC) show that DenTIL has predictive power for response in Nivolumab-treated patients. The model was not predictive of response for the Docetaxel arm. Interestingly, DenTIL was not prognostic of OS in D_6_, in both Docetaxel and Nivolumab-treated patients (see Supplementary Fig. 8).

## Discussion

LUAD and LUSC are two main histological subtypes of NSCLC^33^. Besides the histological differences, LUAD and LUSC have distinct prognosis and, hence, treatment regimens^4,34,35^. Different studies have reported the importance of TILs in clinical outcomes in NSCLC^7,13^. Despite the prognostic value of TIL-based computational image features from H&E in NSCLC, relatively little is known about whether these features are distinct between LUAD and LUSC. This begs the question of whether these TIL-based computational image features reveal different morphologic patterns of TILs that are prognostic of outcome in LUAD and LUSC, respectively. Recent studies have explored the predictive value of immune cell subtypes, particularly T and B cells, in clinical outcomes and treatment response using IHC or QIF^9,36,37^. However, the immune composition of prognostic TIL signatures derived from the H&E images have not been thoroughly studied.

Results of the first experiment showed that the density measures of TILs were prognostic of OS in LUAD, whereas the spatial distribution of TILs was prognostic of OS in LUSC. In LUAD (D_1_), the TIL density measures were statistically significant prognostic of OS on an independent test set (D_1_^test^, *n* = 127) as well as an external validation set (D_5_, *N* = 100). Our findings suggest that TILs are more densely packed as clusters in patients with better survival outcomes as compared to in those with worse outcomes. Results from D_5_ suggest that the DenTIL signature, identified by the model trained on primary resection samples in TCGA-LUAD patients, was associated with clinical outcomes in an external validation set of NSCLC patients treated with a plurality of different neoadjuvant chemotherapeutic regimens. This indicates that the computationally derived signature is robust in capturing biological hallmarks of disease aggressiveness, independent of treatment and tissue sample type.

Results from the external validation set D_6_ suggest that the DenTIL signature, which was prognostic in D_1_, was also predictive of response to treatment in D_6_. This suggests that the model captures signatures related to disease aggressiveness in prediction of both clinical outcome and treatment response. The model showed a signal in predicting therapy response for patients treated with nivolumab but no signal for patients treated with docetaxel. The following are potential reasons as to why the classifier was not predictive of response for the docetaxel-treated patients in D_6_. First, the majority of patients in D_6_ are at stage IV while the model was trained on cohorts with predominantly early-stage tumors; this prominent biological difference might affect the survival impact of TILs. Second, the patients in D_6_ received treatment in the advanced setting where (1) surgery is not a viable option due to metastasis and (2) therapeutic schemes are different from when the treatment is in the neoadjuvant setting (UBern cohort, D_5_) or when the treatment was surgery (TCGA cohort, D_1_ and D_2_); this difference in treatment schemes could have resulted in differential outcomes. Third, the platinum-based therapies used in D_5_ have a different mechanism of action and biological consequence as compared to docetaxel-based therapies used in D_6_. In addition, platinum induces DNA damage and docetaxel alters microtubule formation, which may also contribute to the differences in outcomes. Lastly, the TCGA and UBern cohorts include mostly resected tumors which are relatively larger than the small needle biopsies used in patients with metastatic disease in D_6_, which may have impacted the ability of the model to accurately capture the density and spatial distribution of TILs.

The prognostic ability of TIL density measures is consistent with several previous studies. Corredor et al.^13^. found that DenTIL features were associated with disease recurrence in NSCLC. Other studies have also reported the association between spatial variability of lymphocyte infiltration and poor disease-specific survival^38^ and the association between TIL fraction and OS in various cancer types^18^. In LUSC (D_2_), the most predictive feature was the orientation disorder of TILs, potentially suggesting that TILs in patients at higher risk might be more disorganized as compared to the patients at lower risk. While the prognostic value of cell polarity has not been rigorously shown in NSCLC, it has been shown to be prognostic of progression in pre-invasive breast cancer^39^ and of post-operative biochemical recurrence in prostate cancer^40^. Additionally, the spatial distance between TILs was found to be larger in patients with higher risk as compared to those with lower risk identified by the Kaplan-Meier estimator and log-rank test. The prognostic ability of this graph feature is in line with the study carried out by Wang et al.^41^. The spatial colocalization of TILs and cancer cells was also found to be prognostic of OS, and using these exact features, Corredor et al.^13^ were able to prognosticate recurrence in NSCLC. Taken together, these results reveal distinct spatial morphologic patterns of TILs in LUAD and LUSC. TIL density measures were not prognostic in LUSC; this is in alignment with a study^42^ that reported a negative association between TILs and prognosis in LUSC. Even though previous works explored the association between spatial architecture of TILs and recurrence in NSCLC^13^, our study was different in that we investigated whether the prognostic ability of the spatial patterns is different in LUAD and LUSC. Results from this study indicate the need to build separate TIL-based prognostic models for these subtypes of NSCLC. The prognostic signals from the spatial arrangement of TILs in LUSC also suggest the potential to leverage the spatial attribute of TIL-based biomarkers in prognosis of other squamous cell carcinomas, such as head and neck, cervix, or oral cavity squamous carcinomas. Interestingly, consistency in the prognostic value of morphologic patterns was found across early and late-stage diseases in both LUAD and LUSC (see *Comparison of prognostic morphologic patterns across stages* section in Methods), although these trends need to be further validated in a larger set of late-stage patients.

To evaluate the molecular composition of morphologically distinct TIL signatures in LUAD (D_3_) and LUSC (D_4_), we constructed survival models using TIL features extracted from CD4^+^ T, CD8^+^ T, CD20^+^ B cells, as well as different combinations of these TIL subtypes. In D_1,_ TIL density measures were found to be prognostic of OS in LUAD. In D_3_, density measures of CD4^+^ T cells and of CD8^+^ T cells were both separately found to be prognostic of OS. In a study by Schulze et al.^43^, strong infiltration of CD4^+^ T cells from IHC staining was also found to be associated with improved OS in LUAD. The prognostic value of CD8^+^ T cells is consistent with the study by Schalper et al.^9^, in which they also found CD8^+^ T cells obtained from QIF images to be prognostic of OS in NSCLC independent of other clinical variables. However, our work differed from these previous approaches in that we investigated the spatial distribution of TIL subtypes presented in the images. In this study, we leveraged computational image features to analyze the spatial morphologic patterns of TIL subtypes from QIF stained images. The immune composition of prognostic spatial features of TILs in LUSC (D_4_) was found to be different in comparison with LUAD (D_3_). The spatial interaction between CD4^+^ T and CD8^+^ T cells (e.g., overlapping area of convex hull area of CD4^+^ T and convex hull area of CD8^+^ T cells) and the interaction between CD4^+^ T and CD20^+^ B cells were associated with OS. Specifically, increasing overlapping area between two TIL subtypes was associated with better survival. These results suggest that not only the density of TIL subtypes, but also their spatial colocalization might provide valuable insight into cancer prognosis. Taken together, the results suggest that in LUSC, CD4^+^ T, CD8^+^ T, and CD20^+^ B cells drive the prognostic TIL signatures related to spatial distribution of TILs; whereas in LUAD, TIL density measures of CD4^+^ T and CD8^+^ T cells contribute primarily to prognosis. In support of this, marked differences in immunogenomic contexture and adaptive anti-tumor responses between LUAD and LUSC have been reported^44,45^.

To quantify the presence of TILs or the level of immune infiltration, several approaches such as immunohistochemical (IHC) staining and ssGSEA have been used^46–48^. Several studies have shown the importance of ISs in diagnostic and prognostic tasks in various cancer types^8^. While there has been substantial interest in using quantitative histomorphometric features such as density of immune cells as prognostic biomarkers^10–15,17,18,41^, little is known about whether these features from H&E images are reflective of the ISs derived using ssGSEA. Our findings in Experiment 3 revealed that in both LUAD and LUSC, two of the prognostic TIL features were associated with ISs. In LUAD, two prognostic TIL density measures were associated with ISs and could significantly differentiate low/high IS groups. In general, TIL density is lower in patients with lower ISs as compared to the ones with higher ISs. In LUSC, two of the prognostic graph- and shape-based features were significantly associated with ISs and were significantly differently expressed in low-high IS groups. The pathways that were differentially expressed in the low-high prognostic feature value groups in both LUAD and LUSC are generally related to immune response, immune system process (a process related to development and function of immune system), T cell activation and proliferation, T cell-mediated immunity, and regulation of antigen receptor-mediated signaling pathway (a process that regulates the signaling pathways initiated by cross-linking of antigen receptors on T or B cells) (see Supplementary Fig. 5 for the complete pathways for LUAD and LUSC). Pathways that exist in only LUAD include immune effector process (any process of the immune system contributing to an immune response), T cell-mediated cytotoxicity, B cell proliferation, antigen processing and representation via MHC class lb (antigen-presenting cells with antigen of endogenous origin associated with a MHC non-classical class I molecule protein complex on the cell surface), and positive regulation of I-kappa B Kinase/NF-kappa B signaling (related to regulation of activation and differentiation of innate immune cells and inflammatory T cells)(See Fig. 4 and Supplementary Fig. 5). Finally, the pathways existing only in LUSC include immune response-regulating cell surface receptor signaling pathway (molecular signals related to binding of ligand and receptor on the surface of the target cell capable of activating or suppressing immune response), immune response-activating signal transduction (a series of processes related to change in activities of downstream messengers that eventually lead to activation of immune response), and T cell differentiation. The difference in antigen presentation pathways observed between LUAD and LUSC is in line with the study by McGranahan et al.^44^, where they found that immune-regulatory genes including human lymphocyte antigen class I genes and a component of MHC class I genes had a significantly lower expression in LUSC than in LUAD.

Even though Corredor et al. identified the prognostic value of both spatial and density features of TILs in early-stage NSCLC^13^, the present study is a substantial extension over previous work in that (1) it demonstrated the association of the signature with clinical outcome in an external validation set of NSCLC patients treated with more than six different neoadjuvant chemotherapy regimens, and we further demonstrated the predictive value of the signature in another external validation set of NSCLC patients treated with nivolumab, (2) the prognostic value of spatial and density features were separately investigated in two subtypes of NSCLC to build subtype specific prognostic models, (3) the molecular compositions of prognostic TIL patterns were explored separately in the two subtypes, (4) the association between prognostic features and both immune scores and biological pathways was examined, (5) in survival analysis, Cox proportional hazards regression model^49^, a more robust model that takes into account the effect of risk factors on an outcome over time, was used instead of a binary classifier, (6) WSIs, which can capture the heterogeneity of the immune cells and tumor cells, were used to build the prognostic models instead of TMAs, and (7) the sample size is much larger (*N* > 1000). In addition, while Zhang et al.^35^. found the differences between LUAD and LUSC in terms of genomic alternations and pathways, our work mainly focuses on identifying the differences in terms of computationally-derived imaging features and their biological basis, which was not explicitly covered by Zhang et al. We believe that our quantitative imaging-based approach in interrogating the difference between LUAD and LUSC could complement more expensive gene sequencing, while also offering the benefit of being non-destructive of tissue.

The findings from this study have potential clinical implications. The fact that prognostic patterns of TILs were found to be morphologically and molecularly distinct in LUAD and LUSC suggests the importance of building separate TIL-based prognostic models for the two histologic subtypes. Studies have shown that computer-extracted features of cancer nuclei from H&E images^50^ and spatial arrangement of TILs^20,51,52^ can predict response to immunotherapy in NSCLC and gynecologic cancers respectively. Based on the findings in this study, one may speculate that future predictive models for response to therapy might need to account for the unique morphologic and molecular immune patterns as a function of histologic subtype of NSCLC. Further, given the statistically significant association between computer-extracted TIL features from H&E images and transcriptomics-derived ISs, one may not need to use immunescoring system to prognosticate outcomes. By using routinely acquired diagnostic H&E slides instead of ISs, the approach that involves computationally derived TIL features could not only obviate the need for more expensive gene expression testing and preserve tissue. Additionally, the approaches described in this study might also provide more tailored and specific immune signatures that are respectively predictive for LUAD and LUSC separately; ISs do not account for spatial heterogeneity of immune cell distribution. These features could also be potentially combined with other immunotherapy biomarkers such as PD-L1 immunohistochemistry and tumor mutational burden to enhance patient selection strategies. Furthermore, the method presented in this study could be deployed in future clinical practice to assist clinical decision-making by providing quantitative and objective metrics of response and outcome. This pipeline can be applied as soon as the tissue sample is digitized, so the results could potentially be ready for the pathologists as they review the slides.

Our study has limitations. While the TIL signatures were found to be prognostic of OS in both LUAD and LUSC, their predictive ability such as predicting response to adjuvant chemotherapy or immune checkpoint blockade was not established. In addition, cases included in the analysis were collected retrospectively from multiple institutions and were treated in a non-controlled fashion. The proportion of tissue compartments (epithelial and stromal regions) was not taken into consideration during tile selection and the downstream analysis, which might introduce some bias when comparing the presence of TILs across tiles/patients. Another limitation was the possible introduction selection bias on account of using an automated algorithm to select representative tiles that are similar to each other. In the future, a solution might be to use machine learning approaches to automatically identify the entire tumor region and use all the tiles from that region or to automatically identify tertiary lymphoid structures and extract TIL features from them to predict outcome and response to therapy^53^. We also acknowledge there are other pathways unrelated to immune activities that may also have association with OS in lung cancer, such as angiogenesis, cell differentiation, proliferation, and cell cycling^54^. Future work may benefit from a more comprehensive study analyzing such pathways. Next steps involve addressing these concerns and validating the prognostic signatures from a predictive standpoint by which we will evaluate the ability of these features to predict response to immunotherapy or chemotherapy in late-stage NSCLC.

In summary, we identified unique spatial morphologic TIL signatures that were separately prognostic in LUAD and LUSC based on H&E images, with TIL density measures being prognostic in LUAD and spatial arrangement of TILs being prognostic in LUSC. Further, the prognostic ability of TIL density measures was tested in an external validation set of LUAD patients treated with more than six different neoadjuvant chemotherapy regimens. Using QIF images, we further showed that the immune composition of the morphologically distinct TIL signatures in LUAD and LUSC was different, with CD4^+^ T and CD8^+^ T cells dominating the prognostic signals that capture TIL density measures in LUAD and CD4^+^ T, CD8^+^ T, and CD20^+^ B cells dominating the prognostic signals related to spatial distribution of TILs in LUSC. In both LUAD and LUSC, we discovered associations between prognostic TIL features and ssGSEA-derived ISs. Biological pathways implicated in immune recognition, response, and evasion were significantly differentially expressed with respect to the prognostic features in both LUAD and LUSC. These findings provide a biological basis for computationally derived prognostic measures from TIL patterns on H&E images.

## Methods

### Quality check and preprocessing

Quality check was performed using HistoQC^55^, which provides automated, quantifiable, and reproducible quality control pipelines for detecting artifacts that could potentially compromise image analysis. The H&E slides with suboptimal quality in the dataset were excluded using quality control measures magnification, brightness pen markings, blurriness, and bubbles in the images. For D_1_ and D_2_, inclusion criteria comprised availability of ISs and overall survival information. For patients with multiple H&E slides, the slide with the most abundant tumor tissue area, as determined by our pathologists (P.T., 5 years of experience; P.V., 6 years of experience), was selected for subsequent computational image analysis. For D_3_, D_4_, and D_5_, the inclusion criterion is the availability of overall survival information. For D_6_, the inclusion criteria are the availability of overall survival information, response status, and histologic subtype being LUAD.

**Fig. 6:**
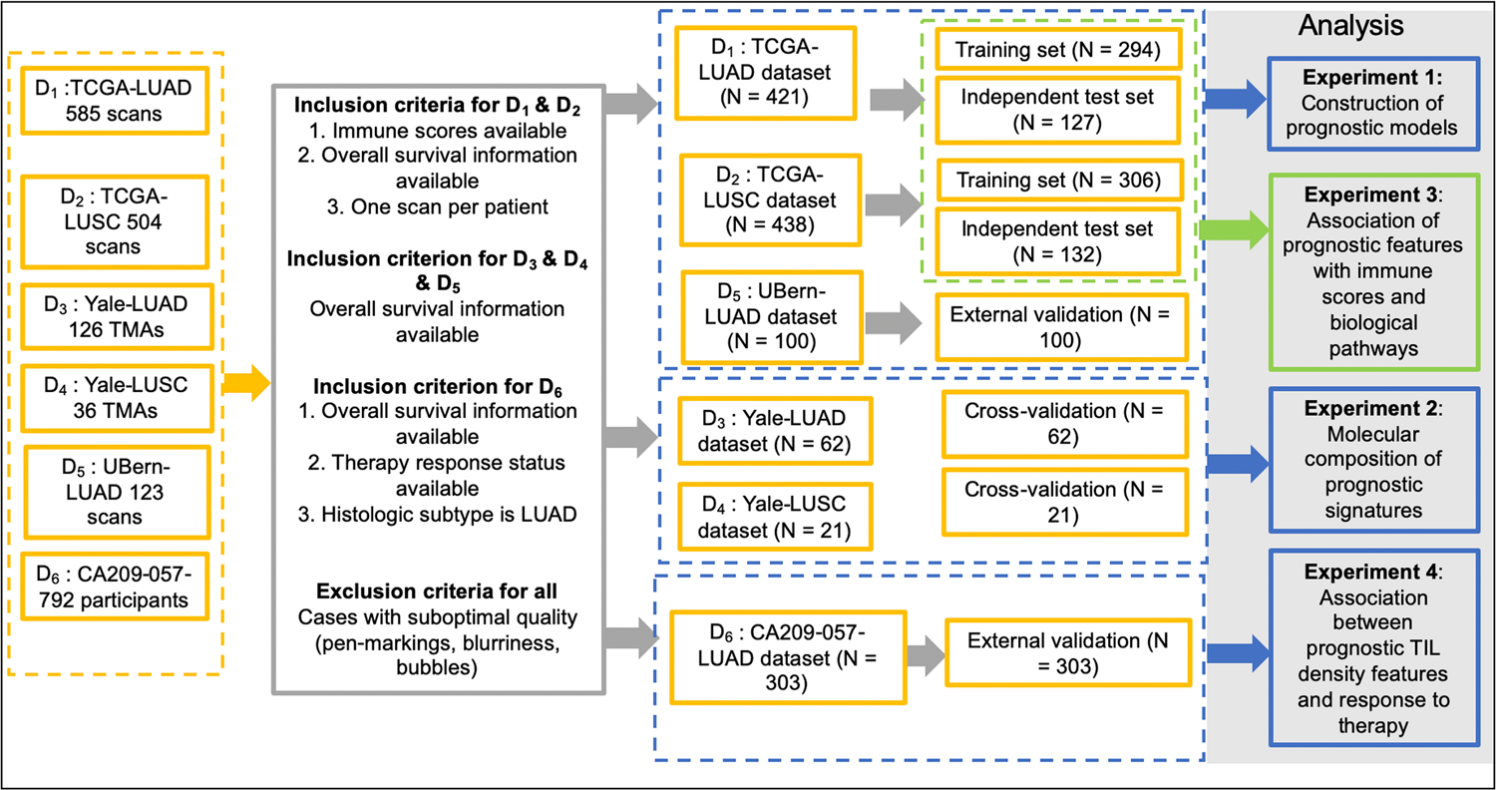
Patient selection workflow for the datasets included in this study. The leftmost column shows the initial datasets. After applying inclusion and exclusion criteria, the final datasets and their corresponding experiments are shown on the right side.

Applying the inclusion and exclusion criteria described above (see Fig. 6) resulted in six datasets for this study, including 421 TCGA-LUAD (D_1_), 438 TCGA-LUSC (D_2_), 62 Yale-LUAD (D_3_), 21 Yale-LUSC (D_4_) cases, 100 UBern-LUAD (D_5_), and 303 CA209-057 (D_6_) cases suitable for the downstream analysis.

### TIL detection and feature extraction

Within the tissue areas on the digitized slide images identified as usable by HistoQC^55^, each WSI was partitioned into smaller non-overlapping tiles of 2048 by 2048 pixels at 0.25 microns per pixel for D_1_, D_2_, and D_6_ and at 0.24 microns per pixel for D_5_. The number of resulting tiles per patient ranged from 18 to 2018 for LUAD, and from 16 to 1393 for LUSC. The individual nuclei on the H&E images were identified and segmented using a watershed-based algorithm^27^ from each of the tiled images. For D_3_ and D_4_, individual nuclei were detected using the same algorithm directly on each digitized TMA spot. The algorithm applies mathematical operations such as fast radial symmetry transformation and regional minima at different scales to detect nuclei. This was followed by a step to distinguish lymphocytes from non-lymphocytes, using the approach reported in Corredor et al.^13^. Briefly, the algorithm involves extracting a series of features relating to the shape, size, and texture of the individual cells in conjunction with a machine learning classifier (a support vector machine with linear kernel) to distinguish TILs from non-TILs. This algorithm was applied to D_1_, D_2_, D_3,_ D_4_, D_5_, and D_6_. Two pathologists (P.T., P.V.) provided a qualitative validation of the nuclei segmentation and TIL detection results.

A targeted tile selection was performed to reduce computational complexity by selecting tiles that preserve the most diversity in D_1_, D_2_, D_5_, and D_6_. Specifically, Principal Component Analysis^56^ was used to reduce the size of the original feature matrix obtained from graph- and shape-based feature extraction (details below), from 240 dimensions to 2 dimensions. Subsequently, kernel density estimation^57^ was used to identify the top 20% most representative tiles corresponding to the kernel density values for each case. Supplementary Fig. 1 shows the kernel density map and example tiles from one patient. These selected tiles were used for the downstream analysis.

19 features relating to the density of TILs (DenTIL features^13^) were extracted. These features include descriptors such as the ratio between the number of TILs and the tissue area, the ratio between the number of TILs and the total number of nuclei, and the ratio between the total area covered by TILs to the total area of the tissue. A total of 350 features to quantify the spatial arrangement of TILs and spatial interaction between TILs and non-TILs (SpaTIL features^13^) were also extracted. These features include area of TIL clusters and the intermixing of the TIL and non-TIL clusters, among others.

240 graph- and shape-based features were extracted from all the nuclei (without distinguishing TILs or non-TILs). These features capture nuclei arrangement using global graph structures^58^ such as minimum spanning tree and Delaunay triangulation, shape features^30^, co-occurring gland tensors (CGTs)^59^, cluster graph features^60^, and cell run-length (CRL) features (See *Cell run-length feature computation* section in Methods). A complete list of features of the density and spatial patterns of TILs, along with other graph- and shape-based features are presented in Tables S2, S3, and S4, respectively.

Features for each case were combined across tiles by calculating statistics such as mean, median, skewness, and kurtosis. For the prognostic analysis, a total of five groups of features were extracted: graph- and shape-based features on all nuclei (960 features), on TILs (960 features), and on non-TILs (960 features), TIL spatial arrangement features (1400 features), and TIL density features (76 features). In case of missing feature values for cluster graph features and CRL features, data imputation^61^ was performed using the average feature values of patients from the same tertile of IS groups. The total number of missing features value was less than one percent.

### Cell Run-length (CRL) feature computation

The quantitative measurements based on local cell graphs have previously been found to be prognostic in oropharyngeal cancers^60^. While most global and local cell graphs measurements are based on a count of the number of vertices/edges, edge lengths, the CRF involves capturing graph complexity by measuring the total number of different ways/runs that the cell graph vertices can be traversed. Steps that comprise the extraction of CRL features are (1) nuclei detection, which was described in the Methods section, (2) nuclei sub-graph construction, (3) CRL computation for each nuclear sub-graph, and (4) CRL feature computation.

For nuclear sub-graph construction, pairwise spatial relationships between cells are defined via localized sub-graphs. A graph *G* = {*V*_*G*_, *E*_*G*_}, where *V*_*G*_ represents the set of *n* nuclear centroids $\gamma _i,\gamma _j \in V_G,i,j \in \{ 1,2, \ldots ,n\}$ as nodes, and *E*_*G*_ represents the set of edges which connect them. The edges between all pairs of nodes *γ*_*i*_, *γ*_*j*_ were computed via the following probabilistic decaying function

1 $$D = \left\{ {\left( {i,j} \right):r \,<\, d\left( {i,j} \right)^{ - \alpha },\forall \gamma _i,\gamma _j \in V} \right\},$$

where *d* (*i*, *j*) represents the Euclidean distance between *γ*_*i*_ and *γ*_*j*_. *α* ≥ 0 controls the density of the graph, where *α* approaching 0 represents a high probability of connecting nodes while αapproaching $\infty$ conversely represents a low probability. $r \in [0,1]$ is an empirically determined edge threshold.

In CRL computation, for every nuclear sub-graph, a cell run-length vector is computed. An example cell graph, comprising 6 cells is shown in Supplementary Fig. 7a, where each vertex represents a cell. A *cell run* is defined as a single traverse from an end point of a cell graph to the other. The *length of run* is defined as the total number of cells being traversed in a run. We denote a length of *n* runs as *n*-runs. In Supplementary Fig. 7a, the cell graph has one 6-run. The corresponding *run length vector* is defined as the number of *cell runs* associated with different lengths of runs.

In the example shown in Supplementary Fig. 7b, the length of runs is numbered from 2 to 6, so that the run-length vector is **R** = [0,0,0,0,1]. Another example of a local cell graph is shown in Supplementary Fig. 7c, in which the cell graph also consists of six cells. However, it contains one 5-run and one 6-run, the corresponding run-length vector is **R** = [0,0,0,1,1]. In terms of the total number of cell runs, the cell graph shown in Supplementary Fig. 7c is more complex compared to the one shown in Supplementary Fig. 7b.

For CRL features computation, we denote the total number of cells in a cell graph as *n*_*c*_, a specific length of run as *r* and its corresponding number of runs as *R*(*r*), the total number of runs in the run-length vector is denoted via $n_r = \mathop {\sum}\limits_r R (r)$. The four features: short-run emphasis *ϕ*_*SRE*_, long-run emphasis *ϕ*_*LRE*_, run-length non-uniformity *ϕ*_*RLN*_, run percentage *ϕ*_*RP*_, extracted from the cell run-length vector are then determined as follows:

2 $$\phi _{SRE} = \frac{1}{{n_r}}\mathop {\sum}\limits_r {\left( {R(r)/r^2} \right)} ,$$

3 $$\phi _{LRE} = \frac{1}{{n_r}}\mathop {\sum}\limits_r {\left( {R(r) \times r^2} \right)} ,$$

4 $$\phi _{RLN} = \frac{1}{{n_r}}\mathop {\sum}\limits_r R (r)^2,$$

and

5 $$\phi _{RP} = \frac{{n_r}}{{n_c}}.$$

The short-run emphasis *ϕ*_*SRE*_ aims to emphasize the shorter cell runs in a cell graph. If one cell graph contains a larger number of shorter runs compared to longer runs, *ϕ*_*SRE*_ will be large. In contrast, the long-run emphasis *ϕ*_*LRE*_ places more importance on longer cell runs compared to shorter ones. If one cell graph contains more long runs compared to shorter ones, *ϕ*_*LRE*_ will be large. The run-length non-uniformity *ϕ*_*RLN*_ is determined by the distribution of cell runs in the run-length vector. It reaches its lower bound while the cell runs are evenly distributed over all run lengths. The run percentage *ϕ*_*RP*_ involves calculating the average cell runs for each cell in the cell graph. If a cell graph has a complex spatial arrangement, the value of *ϕ*_*RP*_ will be large. Since we have several nuclear sub-graphs in any histology image, we employ first-order statistics, mean, standard deviation, kurtosis, skewness, and range, to obtain the final CRL signature for the entire image. Thus, for each image, we have a total of 20 CRL features.

### Co-registration between H&E and QIF images

The subtype assignment for each TIL was challenging due to the imperfect alignment between QIF and H&E images obtained from consecutive tissue sections and the partial overlap of different TIL subtypes on a single cell basis. Different cells have different directions and degrees of misalignment with the corresponding TMA boundaries. Piecewise linear transformation and projective transformation^62^ were used to register the H&E and QIF images. Weighted pixel intensity values of each cell from QIF images were then used to identify the TIL subtype on a per-cell basis. More information on the TIL subtype marker assignment process can be found in Methods under the section *TIL subtype marker assignment process*.

### TIL subtype marker assignment process

The goal was to assign a TIL subtype to each TIL identified from the TMAs. After co-registering QIF images with their corresponding TMA spots, the goal was to identify TIL subtypes on a per-cell basis. This process was based on the comparison of pixel intensities of each cell across images. Specifically, the first step involves getting the pixel intensities around the membrane of each cell where the fluorescent staining is found. Pixel values (0–255) were binned into ten equal-sized segments. Bins belonging to a higher intensity range got higher weights as compared to the bins from a lower intensity range. The product between the number of pixels within a segment and the corresponding weight of that segment was then summed up to be a single value. Then, the ratio between that value and the maximum possible weighted pixel values was obtained. The AQUA value^9^, a quantity used to account for different exposure time of different markers, was then multiplied with the ratio to achieve the intensity correction. By repeating the steps on each cell, we get an intensity corrected final single value for each cell, and the one with the maximum value was assigned to be the true type.

### Statistics

Cox proportional hazards regression model^49^ was built separately for D_1_, D_2_, D_3_, and D_4_ with the LASSO^28^ method as a feature selection tool. After feature selection, a risk threshold was computed to separate the patients into two different risk groups. The risk score *R* of the *i*th patient is defined as $R_i = \mathop {\sum}\nolimits_{i = 1}^n {x_{ij}\beta _j}$, where *x*_*ij*_ is the *j*th feature for the *i*th patient, and *β*_*j*_ is the weight or coefficient of the *j*th selected feature returned by the LASSO method.

The score threshold for dividing patients into low- and high-risk was determined on the training set, and applied to the hold-out testing set of D_1_, D_2_, and the external validation sets D_5_ and D_6_. In training, a three-fold cross validation was used to fit the model. The chosen score threshold was the one yielding statistically significantly different survival risk groups and with the maximum hazard ratio among thresholds yielding a maximum difference in median survival time between the low-risk and high-risk groups. Furthermore, to measure the efficacy of the survival prediction models, the C-Index, log-rank test, HR (95% CI) of each model was evaluated.

Due to the lower sample size for D_3_ (*N* = 62) and D_4_ (*N* = 21), cross-validation was used for the evaluation of the survival risk model and construction of Kaplan-Meier survival estimates^63^ in these two datasets. To evaluate the significance of the log-rank statistic of the cross-validated Kaplan Meier curves, a permutation test^64^ was performed to get the permutation distribution of the log-rank statistic. More details can be found in Methods section *Cross-validated Kaplan-Meier survival curves for QIF dataset*.

### Cross-validated Kaplan-Meier survival curves for QIF dataset

Cross-validation was used for the evaluation of survival risk model and construction of Kaplan-Meier survival curves^63^ on the QIF datasets D_3_ and D_4_ due to the small sample size. In the first iteration of the cross validation (global cross validation), 30% of the patients were randomly selected as the testing set, while the rest of the patients were used for training the survival model. Within the training set of this iteration, the same as in Experiment 1, a threefold cross validation (local cross validation) was used to fit the model. Risk scores were computed as the dot product between selected features and feature weights returned by LASSO. The median risk score was used to separate the patients in the training set into low/high risk groups. In the testing set, patients were divided into low/high risk groups based on the threshold identified from the training set. In the next iteration of the global cross validation, another distinct subset of 30% of the patients (non-overlapping with the subset from the first iteration) were used as a testing set and were divided into low/high risk groups. This global cross validation was repeated until every patient was accounted for in the testing set once and was assigned a risk score. At the end, the low-risk group of the cross-validated Kaplan-Meier curve consisted of all patients classified as low-risk in any of the iteration from the global cross validation, and similarly for the high-risk group.

To evaluate the significance of the log-rank statistic of the cross-validated Kaplan Meier curves, a permutation test was performed to get the permutation distribution of the log-rank statistic^63^. During each iteration of the permutation test, the correspondence of features of different patients was randomly permuted to different survival time and corresponding censoring status. Then the global cross validation was again run using the permuted data. The cross-validated log-rank statistic was then obtained from that permutation test. This was repeated 500 times and the statistical significance level was calculated as the number of times the permuted log-rank statistic was greater than or equal to the un-permuted data. The significance level is an indication of the degree to which survival outcome is independent of the features. The lower the significance level, the more the survival outcome depends on the features.

### Comparison of morphologic patterns across stages

The purpose of this analysis is to check if both the morphologic and molecular differences observed in LUAD and LUSC are consistent across different stages of diseases. To check the consistency of morphologic patterns, the most discriminant features as shown in Supplementary Table 1 of early (stage I and II) and late stage (stage III and IV) patients in LUAD (D_1_) and LUSC (D_2_) were compared using Wilcoxon Rank Sum Test. After applying multiple comparison correction, no statistically significant difference was found across all feature groups except for a graph-based feature in D_2_, the graph average distance to three nearest neighbors of TILs (p = 0.024. See Supplementary Fig. 6). Specifically, patients with late stage disease have higher value for this particular feature, which may suggest that the TILs were more sparsely distributed in those patients. The consistency of molecular differences across different stages in the QIF cohort could not be checked since there were no late-stage patients in LUSC (D_4_).

### Experiment 1: Identifying differences in spatial morphologic patterns of TILs in H&E images between LUAD and LUSC

The purpose of this experiment is to investigate whether the spatial morphologic patterns of TILs from H&E images are potentially different between LUAD and LUSC. To achieve this, five spatial morphologic feature groups, as mentioned in the *Feature Extraction* section, were used to build five distinct prognostic models independently in LUAD and LUSC. For each of the five prognostic models in D_1_ and D_2_ respectively, 70% of the cases were randomly subsampled to be in the training set, and the remaining 30% were in the independent testing set. This resulted in 294 patients in the training set (D_1_^train^) and 127 patients in the independent testing set (D_1_^test^) for D_1_, and 306 patients in the training set (D_2_^train^) and 132 patients in the independent testing set (D_2_^test^) for D_2_. The model trained using D_1_ (feature weights and risk threshold) was also directly applied to the external validation sets D_5_ and D_6_. Features were normalized to a range between 0 and 1, and the same normalization mapping was applied to the test set and the external validation set.

### Experiment 2: Identifying differences in molecular composition of prognostic TIL signatures between LUAD and LUSC using QIF images

We sought to further investigate whether the TIL signatures found to be prognostic in D_1_ and D_2_ are distinct in their immune composition using the QIF images in D_3_ and D_4_. In other words, the purpose of Experiment 1 was to identify a set of prognostic features, and the purpose of Experiment 2 was to investigate the cell composition of the features associated with clinical outcome identified from Experiment 1. The rationale for this experiment was that by comparing the performance of these different survival models across LUAD (D_3_) and LUSC (D_3_), one could then speculate the potential differences in the molecular composition of the overall TIL signatures found to be prognostic in LUAD (D_1_) and LUSC (D_2_). To achieve this, groups of spatial morphologic features found to be prognostic of OS in D_1_ and D_2_ were extracted from a total of six groups of TIL subtypes and combinations of TIL subtypes presented in D_3_ and D_4_, and a new set of survival models were trained based on these features extracted from D_3_ and D_4_.

Specifically, TIL density measures and graph- and shape-based features were extracted from (1) CD4^+^ T cells only, (2) CD8^+^ T cells only, (3) CD20^+^ B cells only, (4) both CD4^+^ T cells and (plus) CD8^+^ T cells, (5) both CD4^+^ T cells and CD20^+^ B cells, and (6) both CD8^+^ T cells and CD20^+^ B cells. SpaTIL features which capture the interaction between 2 groups of cells were also extracted using the following pairs of cells: (1) CD4^+^ T cells as group 1 versus the rest of cells as group 2, (2) CD8^+^ T cells as group 1 versus the rest of cells as group 2, (3) CD20^+^ B cells as group 1 versus the rest of cells as group 2, (4) CD4^+^ T cells as group 1 versus CD8^+^ T cells as group 2, (5) CD4^+^ T cells as group 1 versus CD20^+^ B cells as group 2, and (6) CD8^+^ T cells as group 1 versus CD20^+^ B cells as group 2.

### Experiment 3: Association of prognostic features from H&E images with ISs and biological pathways

The ISs used in this study (for D_1_ and D_2_) were obtained from the ESTIMATE algorithm presented by Yoshihara et al.^29^, where an immune signature was used to capture the presence of infiltration of immune cells in the tumor region. To generate these signatures, immune-cell related gene signatures were obtained using the overlap between gene expression profiles of normal hematopoietic genes and those of other normal cell types. ssGSEA was then used to calculate the ISs from the immune signatures to capture the presence of immune cells in tumor tissues. The ESTIMATE algorithm was applied to multiple disease types from the TCGA datasets, including LUAD and LUSC. ISs have been shown to be prognostic of outcomes, associated with immune response and treatment response in various cancer types such as breast cancer^8^, melanoma^8^, clear cell renal cell carcinoma^65^, and metastatic colorectal cancer^66^. Using ESTIMATE scores, Meng et al.^67^ identified genes related to the regulation of immune response of pancreatic adenocarcinoma patients. Khorrami et al. found the positive association between the existence of TILs and response to immunotherapy^68^. An immune scoring system surrogating for composition of tumor and immune cells may allow prediction of response to immunotherapy, and thereby help in identification of suitable candidates for therapy administration. To investigate whether features prognostic of OS were also associated with ISs, we computed the pairwise Spearman correlation coefficients accounting for multiple statistical inferences^69^ between each selected feature and IS of the patients between the prognostic features and IS in D_1_ and D_2_.

Normalized mRNA sequencing data obtained from TCGA was available for all 421 LUAD and 438 LUSC patients in D_1_ and D_2_ in our study. The dataset, consisting of 20,531 genes, was used to investigate the underlying biological pathways associated with the prognostic features obtained from the digitized tissue slides. First, a Spearman correlation coefficient along with p-value was calculated between each of the 20,531 gene expression scores and the risk scores from the survival analysis. This process resulted in genes that were significantly associated with the risk scores derived from prognostic models. In the GO analysis platform^31,32^, these genes were then used to identify biological pathways associated with the risk scores after accounting for the false discovery rate (FDR)^69^ of 0.05. After obtaining the overrepresented genes from the biological pathways, ssGSEA was performed to get the enrichment scores. The median value of the prognostic features from the LASSO method was used to obtain two groups of patients with low and high expression value of the features. Wilcoxon Rank Sum Test was then used to investigate whether there is a statistically significant difference in the distribution of the enrichment scores in the low-high feature value groups, after controlling for FDR^69^.

### Experiment 4: Association between prognostic TIL density features and response to therapy

After applying the risk threshold obtained from the training set D_1_, the patients in D_6_ are predicted as being at either low or high risk. In the context of associating Cox-model predicted survival risk and the response to therapy, a true positive is when a patient is predicted as having high-risk and is a non-responder; a true negative is when a patient is predicted as having low-risk and is a responder; a false positive is when a patient is predicted as having high-risk and is a responder; a false negative is when a patient is predicted as having low-risk and is a non-responder. The AUC curves can then be generated based on the confusion matrix formed by the predicted risk category and response status.

### Reporting summary

Further information on research design is available in the Nature Research Reporting Summary linked to this article.

## Supplementary information

Supplementary information

REPORTING SUMMARY

## Data Availability

The D1 (TCGA-LUAD)^22^ and D2 (TCGA-LUSC)^23^ datasets were generated by TCGA Research Network (http://cancergenome.nih.gov/), and are deposited in dbGAP under accession phs000178. The D3 (Yale-LUAD)^9^, D4 (YALE-LUSC)^24^, D5 (UBern-LUAD)^25^, and D6 (CA209-057)^26^ include data that are protected through institutional compliance. The clinical repository of cases can only be shared per specific institutional review board (IRB) requirements. Upon reasonable request to each respective author/institution, a data sharing agreement can be initiated between the interested parties and the clinical institution following institution-specific guidelines.

## Code availability

The code is available at: (1) https://github.com/gercop2000/SpaTIL, (2) https://github.com/gercop2000/DenTIL.

## References

[1] Ferlay J (2015). Cancer incidence and mortality worldwide: sources, methods and major patterns in GLOBOCAN 2012. Int. J. Cancer.

[2] Molina JR, Yang P, Cassivi SD, Schild SE, Adjei AA (2008). Non-small cell lung cancer: epidemiology, risk factors, treatment, and survivorship. Mayo Clin. Proc..

[3] Lu T (2019). Trends in the incidence, treatment, and survival of patients with lung cancer in the last four decades. Cancer Manag. Res..

[4] Wang BY (2020). The comparison between adenocarcinoma and squamous cell carcinoma in lung cancer patients. J. Cancer Res. Clin. Oncol..

[5] Scagliotti G (2009). The differential efficacy of pemetrexed according to NSCLC histology: a review of two phase III studies. Oncologist.

[6] Seo JS, Kim A, Shin JY, Kim YT (2018). Comprehensive analysis of the tumor immune micro-environment in non-small cell lung cancer for efficacy of checkpoint inhibitor. Sci. Rep..

[7] Bremnes RM (2016). The role of tumor-infiltrating lymphocytes in development, progression, and prognosis of non-small cell lung cancer. J. Thorac. Oncol..

[8] Liu W (2018). Transcriptome-derived stromal and immune scores infer clinical outcomes of patients with cancer. Oncol. Lett..

[9] Schalper KA (2015). Objective measurement and clinical significance of TILs in non-small cell lung cancer. J. Natl Cancer Inst..

[10] Brambilla E (2016). Prognostic effect of tumor lymphocytic infiltration in resectable non-small-cell lung cancer. J. Clin. Oncol..

[11] Bremnes RM, Donnem T, Busund LT (2016). Importance of tumor infiltrating lymphocytes in non-small cell lung cancer?. Ann. Transl. Med..

[12] Brown JR (2014). Multiplexed quantitative analysis of CD3, CD8, and CD20 predicts response to neoadjuvant chemotherapy in breast cancer. Clin. Cancer Res..

[13] Corredor G (2019). Spatial architecture and arrangement of tumor-infiltrating lymphocytes for predicting likelihood of recurrence in early-stage non–small cell lung cancer. Clin. Cancer Res..

[14] Kashiwagi S (2017). Use of tumor-infiltrating lymphocytes (TILs) to predict the treatment response to eribulin chemotherapy in breast cancer. PLoS One.

[15] Kochi M (2018). Tumour-infiltrating lymphocytes (TILs)-related genomic signature predicts chemotherapy response in breast cancer. Breast Cancer Res. Treat..

[16] Wang K, Xu J, Zhang T, Xue D (2016). Tumor-infiltrating lymphocytes in breast cancer predict the response to chemotherapy and survival outcome: a meta-analysis. Oncotarget.

[17] AbdulJabbar K (2020). Geospatial immune variability illuminates differential evolution of lung adenocarcinoma. Nat. Med..

[18] Saltz J (2018). Spatial organization and molecular correlation of tumor-infiltrating lymphocytes using deep learning on pathology images. Cell Rep..

[19] Bruni, D., Angell, H. K. & Galon, J. The immune contexture and Immunoscore in cancer prognosis and therapeutic efficacy. *Nat. Rev. Cancer* 1–19 10.1038/s41568-020-0285-7 (2020). 10.1038/s41568-020-0285-7 32753728

[20] Vaidya P (2020). CT derived radiomic score for predicting the added benefit of adjuvant chemotherapy following surgery in stage I II resectable non-small cell lung cancer: a retrospective multicohort study outcome prediction. Lancet Digit. Heal..

[21] Salgado R (2015). The evaluation of tumor-infiltrating lymphocytes (TILS) in breast cancer: recommendations by an International TILS Working Group 2014. Ann. Oncol..

[22] Collisson EA (2014). Comprehensive molecular profiling of lung adenocarcinoma: the cancer genome atlas research network. Nature.

[23] Hammerman PS (2012). Comprehensive genomic characterization of squamous cell lung cancers. Nature.

[24] Camp RL, Chung GG, Rimm DL (2002). Automated subcellular localization and quantification of protein expression in tissue microarrays. Nat. Med..

[25] Keller MD (2018). Adverse prognostic value of PD-L1 expression in primary resected pulmonary squamous cell carcinomas and paired mediastinal lymph node metastases. Mod. Pathol..

[26] Borghaei H (2015). Nivolumab versus docetaxel in advanced nonsquamous non-small-cell lung cancer. N. Engl. J. Med..

[27] Veta M (2013). Automatic nuclei segmentation in H&E stained breast cancer histopathology images. PLoS One.

[28] Tibshirani R (1997). The lasso method for variable selection in the cox model. Stat. Med..

[29] Yoshihara K (2013). Inferring tumour purity and stromal and immune cell admixture from expression data. Nat. Commun..

[30] Zhang D, Lu G (2002). Shape-based image retrieval using generic Fourier descriptor. Signal Process. Image Commun..

[31] Ashburner M (2000). Gene ontology: tool for the unification of biology. Nat. Genet..

[32] Carbon S (2017). Expansion of the gene ontology knowledgebase and resources: the gene ontology consortium. Nucleic Acids Res..

[33] Ho C, Tong KM, Ramsden K, Ionescu DN, Laskin J (2015). Histologic classification of non-small-cell lung cancer over time: reducing the rates of not-otherwise-specified. Curr. Oncol..

[34] Huang T (2016). Distinguishing lung adenocarcinoma from lung squamous cell carcinoma by two hypomethylated and three hypermethylated genes: a meta-analysis. PLoS One.

[35] Zhang XC (2019). Comprehensive genomic and immunological characterization of Chinese non-small cell lung cancer patients. Nat. Commun..

[36] Patel AJ, Richter A, Drayson MT, Middleton GW (2020). The role of B lymphocytes in the immuno-biology of non-small-cell lung cancer. Cancer Immunol. Immunother..

[37] Taube JM (2018). Implications of the tumor immune microenvironment for staging and therapeutics. Mod. Pathol..

[38] Khan AM, Yuan Y (2016). Biopsy variability of lymphocytic infiltration in breast cancer subtypes and the ImmunoSkew score. Sci. Rep..

[39] Li H (2019). Quantitative nuclear histomorphometric features are predictive of oncotype DX risk categories in ductal carcinoma in situ: preliminary findings. Breast Cancer Res..

[40] Lee G (2017). Nuclear shape and architecture in benign fields predict biochemical recurrence in prostate cancer patients following radical prostatectomy: preliminary findings. Eur. Urol. Focus.

[41] Wang, X. et al. Prediction of recurrence in early stage non-small cell lung cancer using computer extracted nuclear features from digital H&E images OPEN. 10.1038/s41598-017-13773-7. 10.1038/s41598-017-13773-7 PMC5648794 29051570

[42] Nirmal AJ (2018). Immune cell gene signatures for profiling the microenvironment of solid tumors. Cancer Immunol. Res..

[43] Schulze AB (2020). Tumor infiltrating T cells influence prognosis in stage I-III non-small cell lung cancer. J. Thorac. Dis..

[44] McGranahan N (2016). Clonal neoantigens elicit T cell immunoreactivity and sensitivity to immune checkpoint blockade. Science.

[45] Gettinger SN (2018). A dormant TIL phenotype defines non-small cell lung carcinomas sensitive to immune checkpoint blockers. Nat. Commun..

[46] Hofman P (2019). Multiplexed immunohistochemistry for molecular and immune profiling in lung cancer—just about ready for prime-time?. Cancers.

[47] Gentles AJ (2015). The prognostic landscape of genes and infiltrating immune cells across human cancers. Nat. Med..

[48] Danaher P (2017). Gene expression markers of tumor infiltrating leukocytes. J. Immunother. Cancer.

[49] Fisher LD, Lin DY (1999). Time-dependent covariates in the Cox proportional-hazards regression model. Annu. Rev. Public Health.

[50] Wang X (2018). Computer extracted features of cancer nuclei from H&E stained tissues of tumor predicts response to nivolumab in non-small cell lung cancer. J. Clin. Oncol..

[51] Barrera C (2018). Computer-extracted features relating to spatial arrangement of tumor infiltrating lymphocytes to predict response to nivolumab in non-small cell lung cancer (NSCLC). J. Clin. Oncol..

[52] Azarianpour, S. et al. Computer extracted features related to the spatial arrangement of tumor-infiltrating lymphocytes predict overall survival in epithelial ovarian cancer. in: *Medical Imaging 2020: Digital Pathology* (Tomaszewski, J. E. & Ward, A. D. eds.) **11320**, 25 (SPIE, 2020).

[53] Sautès-Fridman C, Petitprez F, Calderaro J, Fridman WH (2019). Tertiary lymphoid structures in the era of cancer immunotherapy. Nat. Rev. Cancer.

[54] Herbst RS, Morgensztern D, Boshoff C (2018). The biology and management of non-small cell lung cancer. Nature.

[55] Janowczyk, A., Zuo, R., Gilmore, H., Feldman, M. & Madabhushi, A. HistoQC: an open-source quality control tool for digital pathology slides. *JCO Clin. Cancer Inform.* 1–7 10.1200/cci.18.00157 (2019). 10.1200/CCI.18.00157 PMC6552675 30990737

[56] Wold S, Esbensen K, Geladi P (1987). Principal component analysis. Chemom. Intell. Lab. Syst..

[57] Terrell, G. R. & Scott, D. W. Variable kernel density estimation. http://www.jstor.org/stable/2242011. Ann. Statist. **20**, 1236–1265 (1992).

[58] Lee, G. C. An integrated companion diagnostics assay for predicting biochemical recurrence following radical prostatectomy. Semantic Scholar. https://www.semanticscholar.org/paper/An-integrated-companion-diagnostics-assay-for-Lee/4a8b7dee1adc09560c59fdaf7b048fa3dcc997a9?p2df (2014) (Accessed 15 Oct 2020).

[59] Lee, G. et al. Cell Orientation Entropy (COrE): predicting biochemical recurrence from prostate cancer tissue microarrays. in *Lecture Notes in Computer Science (including subseries Lecture Notes in Artificial Intelligence and Lecture Notes in Bioinformatics)* **8151** LNCS, 396–403 (Springer, Berlin, Heidelberg, 2013). 10.1007/978-3-642-40760-4_50 24505786

[60] Ali, S., Veltri, R., Epstein, J. A., Christudass, C. & Madabhushi, A. Cell cluster graph for prediction of biochemical recurrence in prostate cancer patients from tissue microarrays. in *Medical Imaging 2013: Digital Pathology* (Gurcan, M. N. & Madabhushi, A. eds) 8676, 86760H (SPIE, 2013).

[61] Efron B (1994). Missing data, imputation, and the bootstrap. J. Am. Stat. Assoc..

[62] Goshtasby A (1986). Piecewise linear mapping functions for image registration. Pattern Recognit..

[63] Simon RM, Subramanian J, Li MC, Menezes S (2011). Using cross-validation to evaluate predictive accuracy of survival risk classifiers based on high-dimensional data. Brief Bioinform..

[64] Verweij PJM, Van Houwelingen HC (1993). Cross‐validation in survival analysis. Stat. Med..

[65] Xu WH (2019). Prognostic value and immune infiltration of novel signatures in clear cell renal cell carcinoma microenvironment. Aging.

[66] Zhou R (2019). Immune cell infiltration as a biomarker for the diagnosis and prognosis of stage I–III colon cancer. Cancer Immunol. Immunother..

[67] Meng Z (2020). Using ESTIMATE algorithm to establish an 8-mRNA signature prognosis prediction system and identify immunocyte infiltration-related genes in Pancreatic adenocarcinoma. Aging.

[68] Khorrami M (2020). Changes in CT radiomic features associated with lymphocyte distribution predict overall survival and response to immunotherapy in non-small cell lung cancer. Cancer Immunol. Res..

[69] Benjamini Y, Hochberg Y (1995). Controlling the false discovery rate: a practical and powerful approach to multiple. Test. J. R. Stat. Soc. Ser. B.

