# Supplementary material for: Image analysis reveals molecularly distinct patterns of TILs in NSCLC associated with treatment outcome
Source: NPJ Precis Oncol. 2022 Jun 3;6:33. doi: 10.1038/s41698-022-00277-5 (PMC9166700; doi:10.1038/s41698-022-00277-5)
Supplement: Supplementary file 1 — Supplementary information [file 41698_2022_277_MOESM1_ESM.pdf]

Supplementary Information

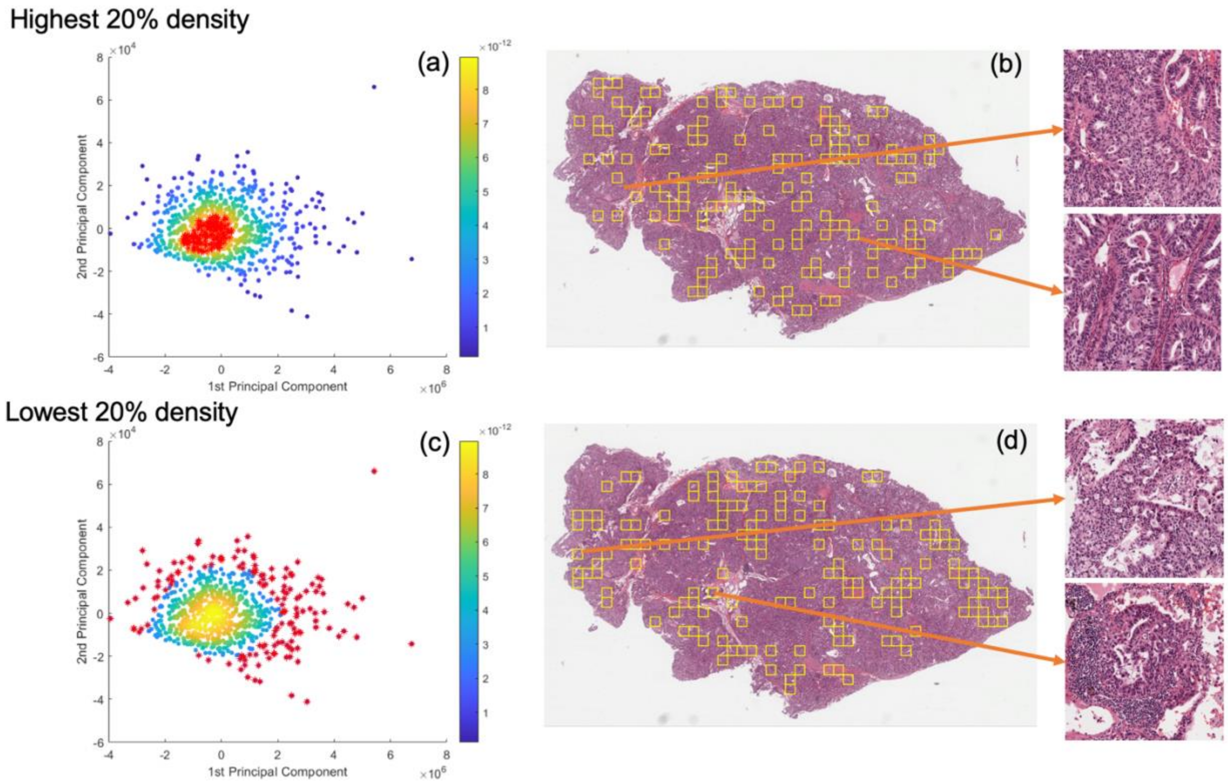

Supplementary Figure 1. Visualization of PCA and KDE from a LUAD case in D_1. (a) and (c) show the kernel density maps with each point representing one image tile. In (a) and (c), the points from the highest and lowest 20% density regions, respectively, are highlighted by overlaying red markers on the originally colored dots from the colormap. (b) and (d) show the tile locations highlighted in yellow squares for highest density region (figure b) and lowest density region (figure d) tiles.

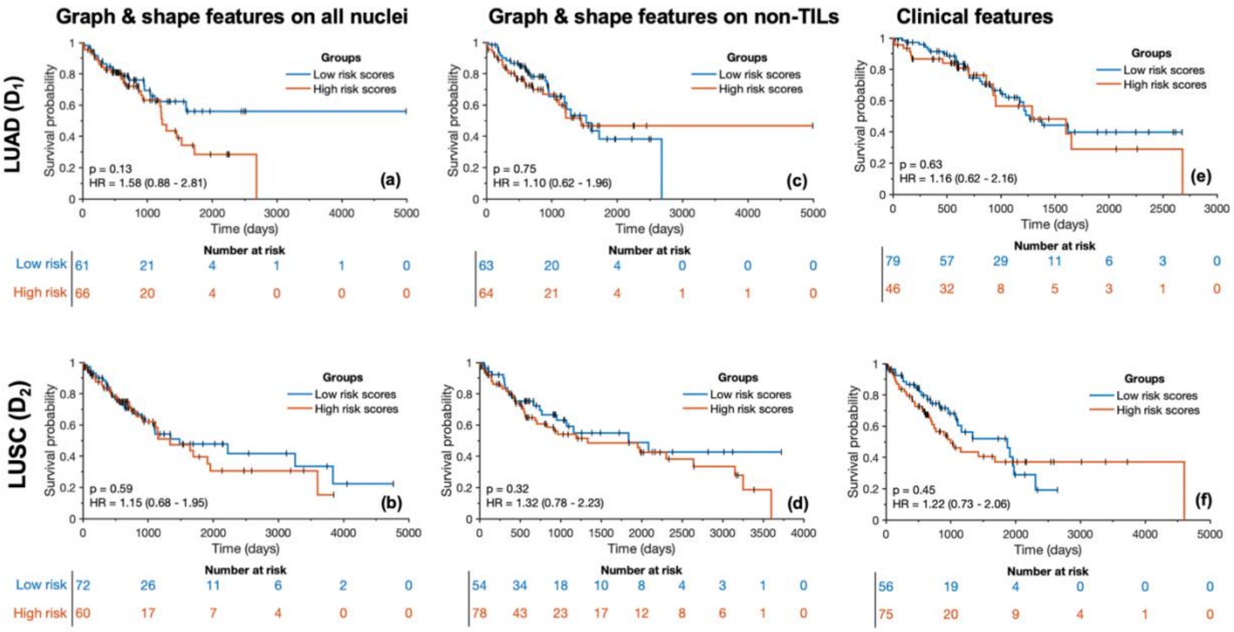

Supplementary Figure 2. The Kaplan-Meier (KM) survival curves generated from features extracted from all nuclei and non-TILs, as well as clinical features, on independent test set of D_1 and D_2. The top row represents the KM curves of LUAD cases in D_1 and the bottom row represents those for LUSC cases in D_2. (a) and (b) represent the graph/shape features on all nuclei, (c) and (d) represent the graph/shape features on non-TILs, and (e) and (f) represent clinical features which include gender, age, tumor stage and cigarettes per day.

A Cross-validated Kaplan-Meier survival curves using density features on TIL subtypes in LUAD

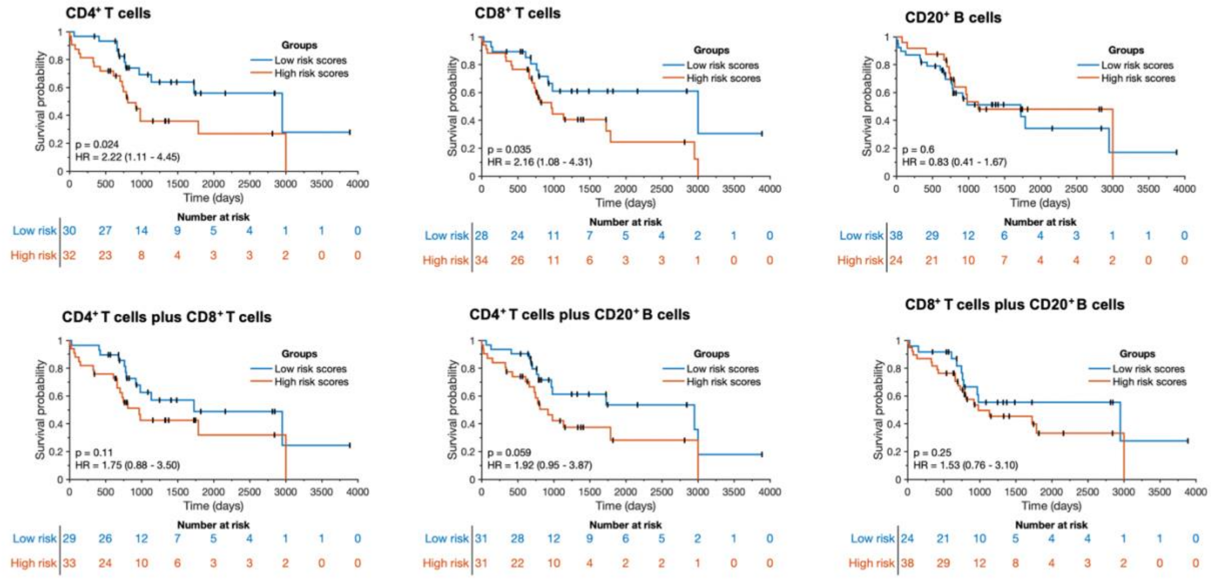

B Cross-validated Kaplan-Meier survival curves using spatial arrangement features in TIL subtypes in LUSC

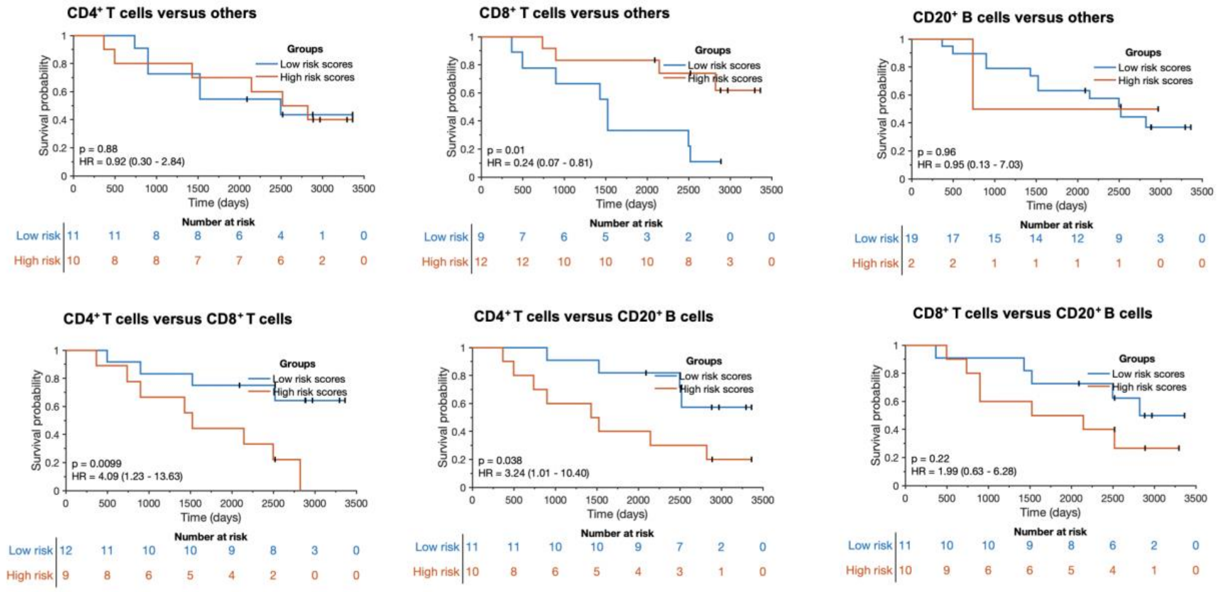

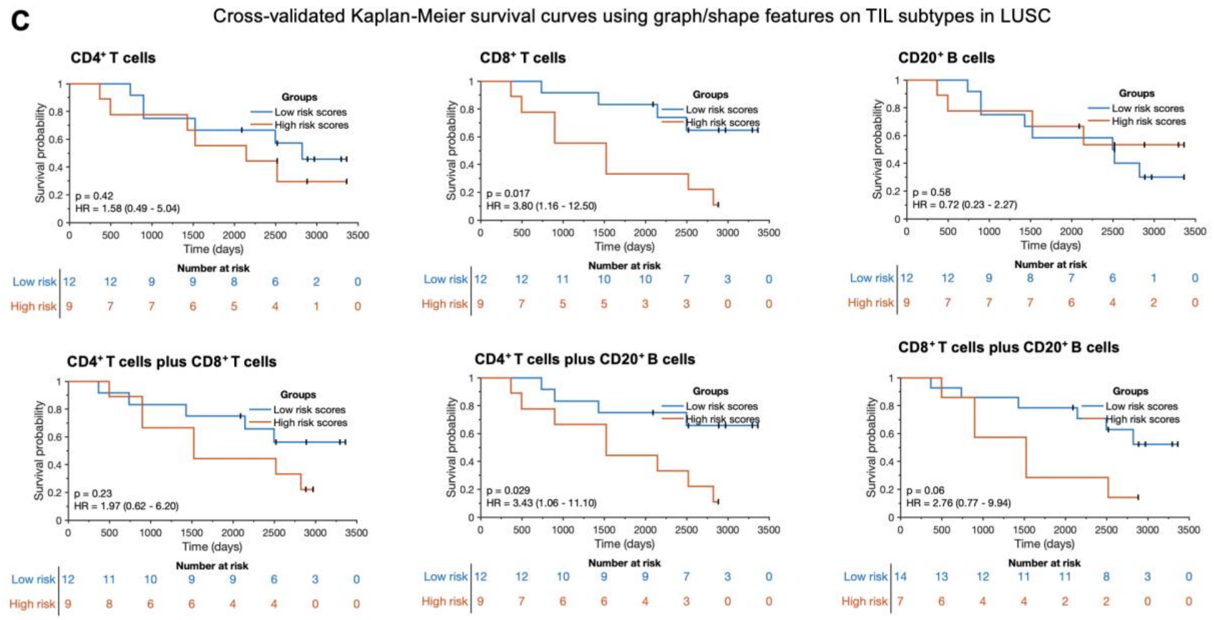

Supplementary Figure 3. The cross-validated Kaplan-Meier (KM) survival curves on different features extracted from different TIL subtypes in D_3 and D_4. *A* represents the KM curves of density measures of TIL subtypes in LUAD from D_3, *B* represents the spatial interaction between TIL subtypes in LUSC from D_4, and *C* represents the graph and shape features on TIL subtypes in LUSC from D_4.

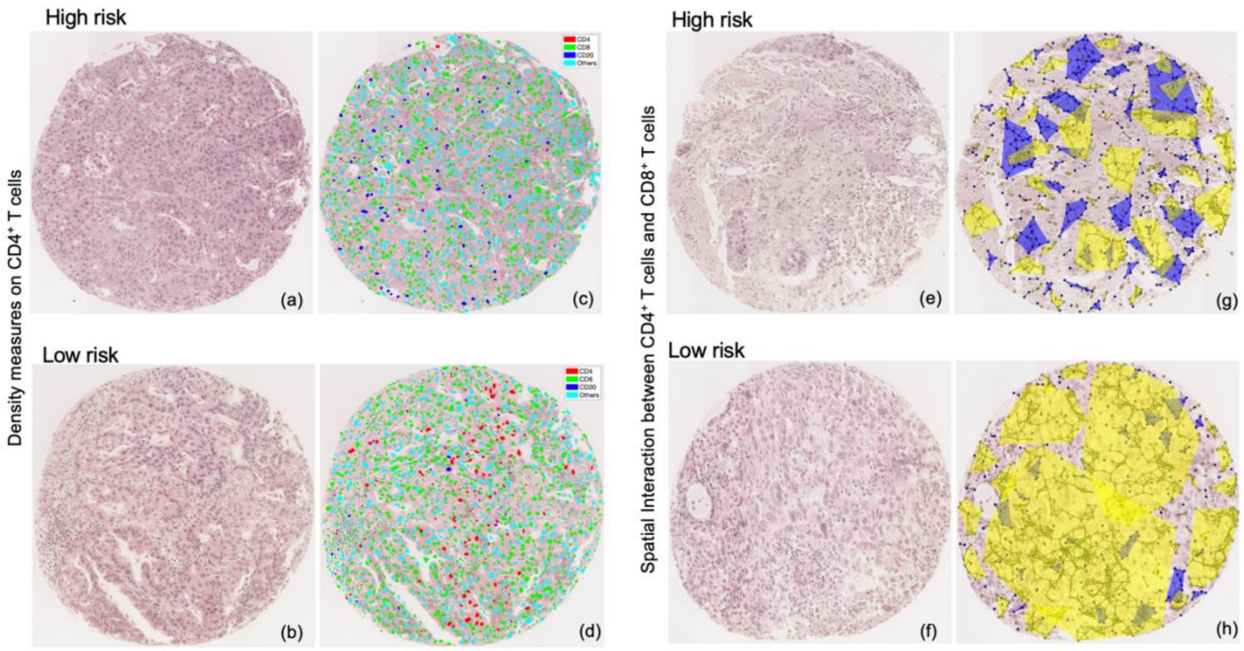

Supplementary Figure 4. Visualization of computational image features on different immune cell subtypes from datasets D_3 and D_4. (a)-(d) represent the visualization of density measures of $CD4^+$ T cells. (a) and (b) show the TMAs of a high-risk and low-risk case, respectively. (c) and (d) show the corresponding TIL subtype maps highlighting $CD4^+$ T cells in red, $CD8^+$ T cells in green, $CD20^+$ B cells in blue, and all other cell types in cyan. $CD4^+$ T cells density is higher in the low-risk patient as compared to the high-risk patient. (e)-(h) show the spatial colocalization of $CD4^+$ T cells and $CD8^+$ T cells. (e) and (f) show the TMAs of a high-risk and low-risk case respectively. (g) and (h) show the corresponding feature maps with convex hull of $CD4^+$ T cells (purple) and $CD8^+$ T cells (yellow). The average area of CD8 convex hull (clusters), and the intersected area between CD4 and CD8 clusters are both larger in the low-risk patient than in the high-risk patient.

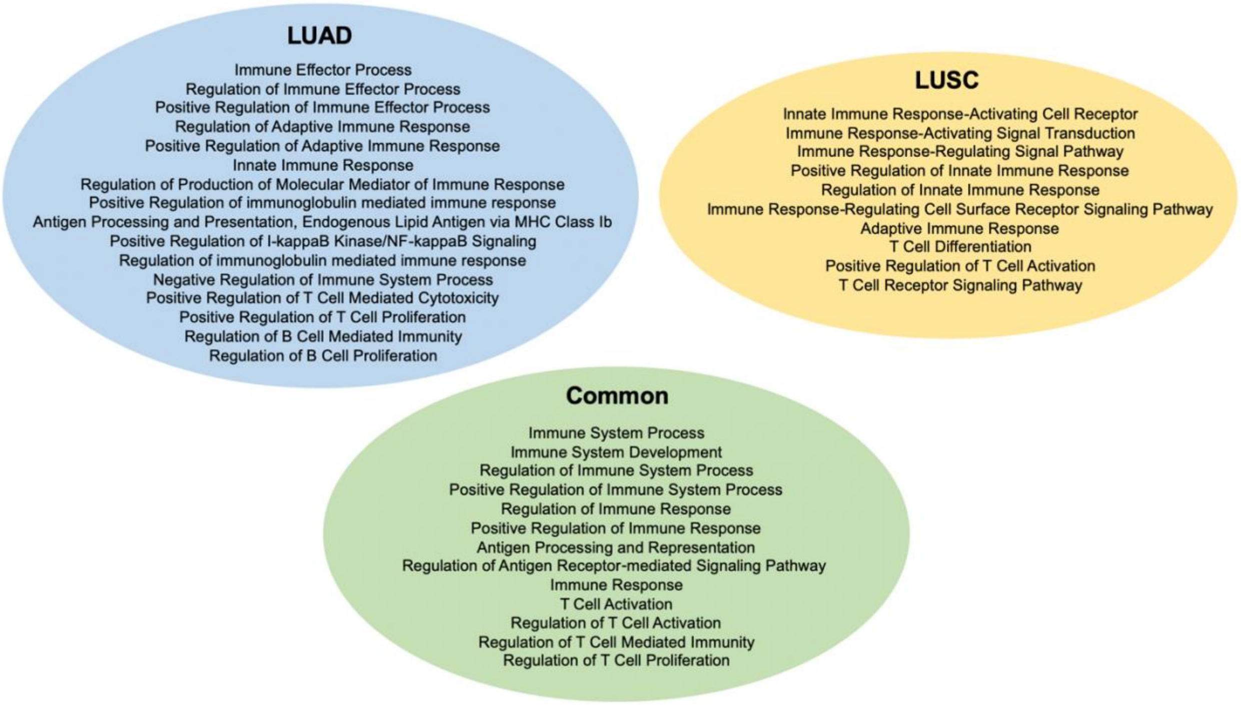

Supplementary Figure 5. The biological pathways implicated in immune response that were significantly differentially expressed in the two patient groups with low and high values of the prognostic features. The common pathways shared between LUAD and LUSC are within the green circle. The pathways unique to LUAD are within the blue circle, and those unique to LUSC are within the yellow circle.

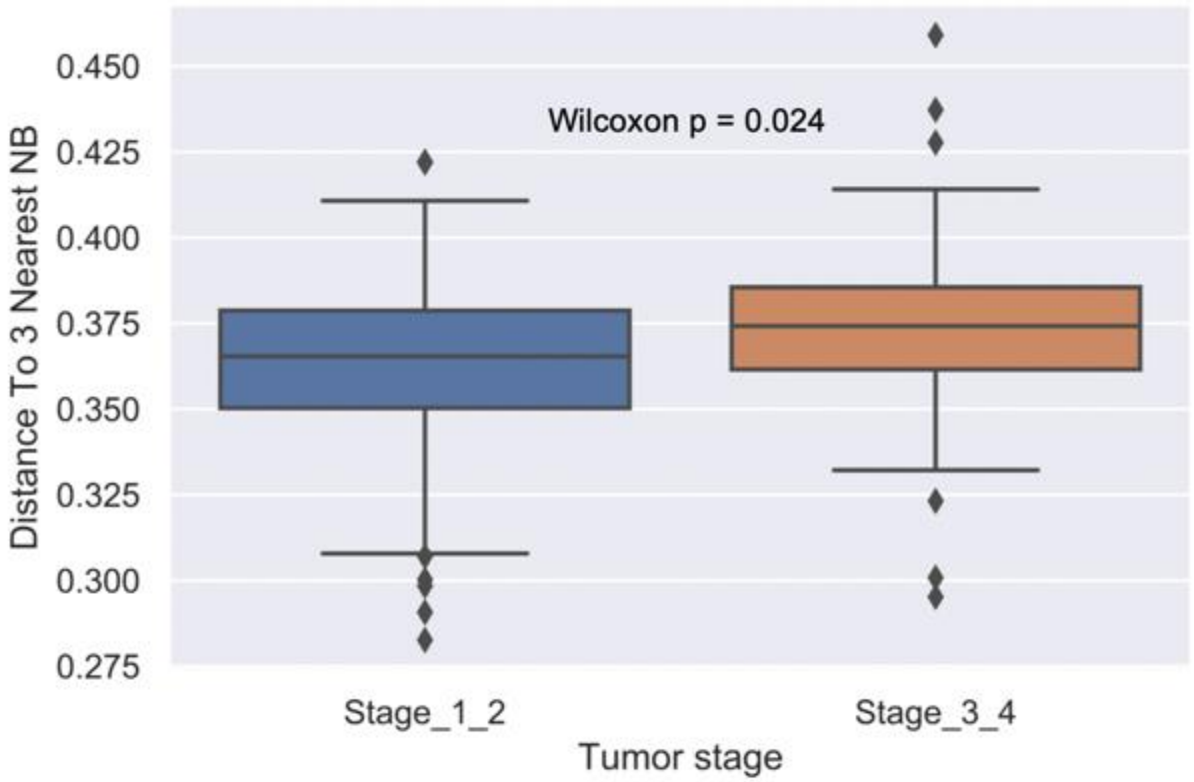

Supplementary Figure 6. The distribution of one of the discriminant graph-based features, graph average of distance to three nearest neighbors of TILs, in early and late stage cases in D_2.

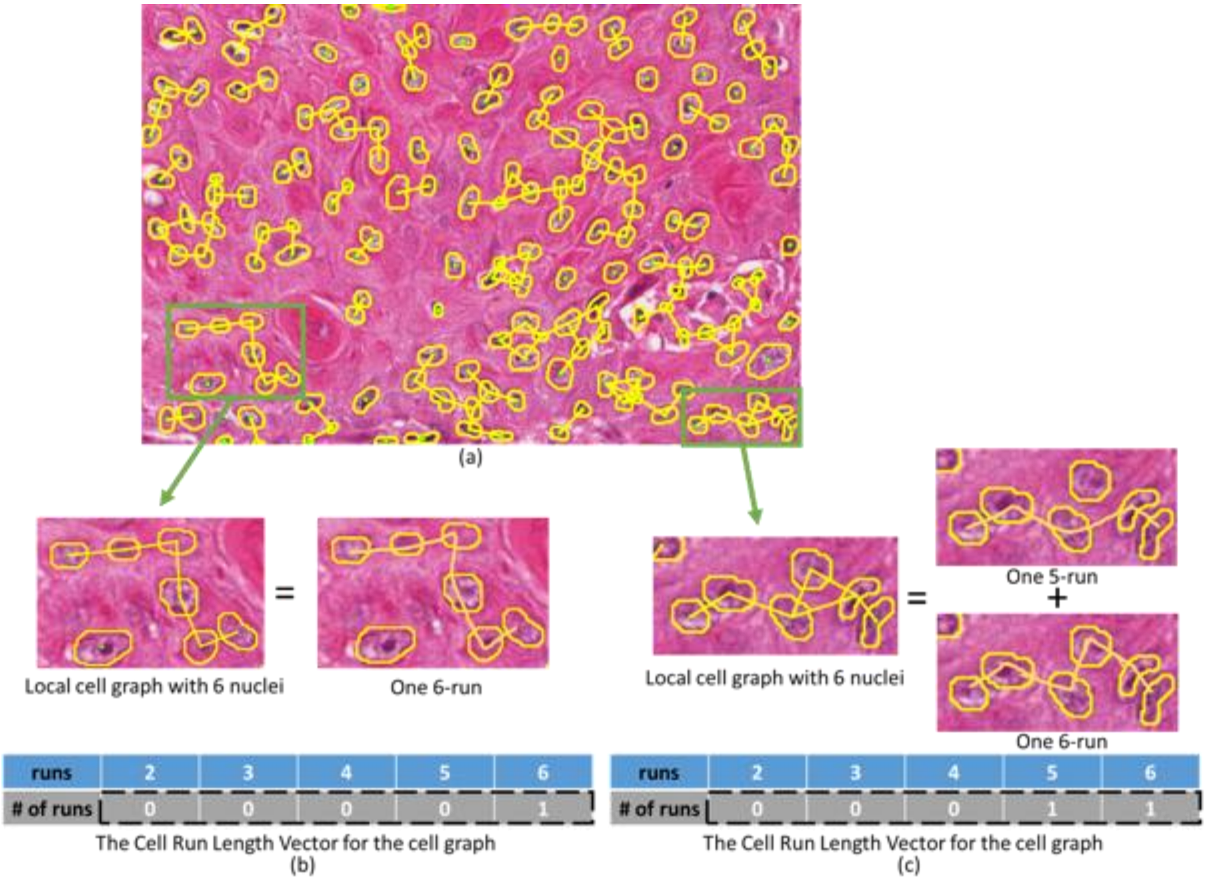

Supplementary Figure 7. Illustration of the basic concept of cell run-length graph with H&E image examples. (a) shows the original H&E image with pre-segmented nuclear contours and corresponding local cell graphs. Two typical local cell graphs (both 6-cell cliques) are shown in (b) and (c), respectively. In (c) the cell graph is decomposed into separate cliques comprising one 5-run and one 6-run.

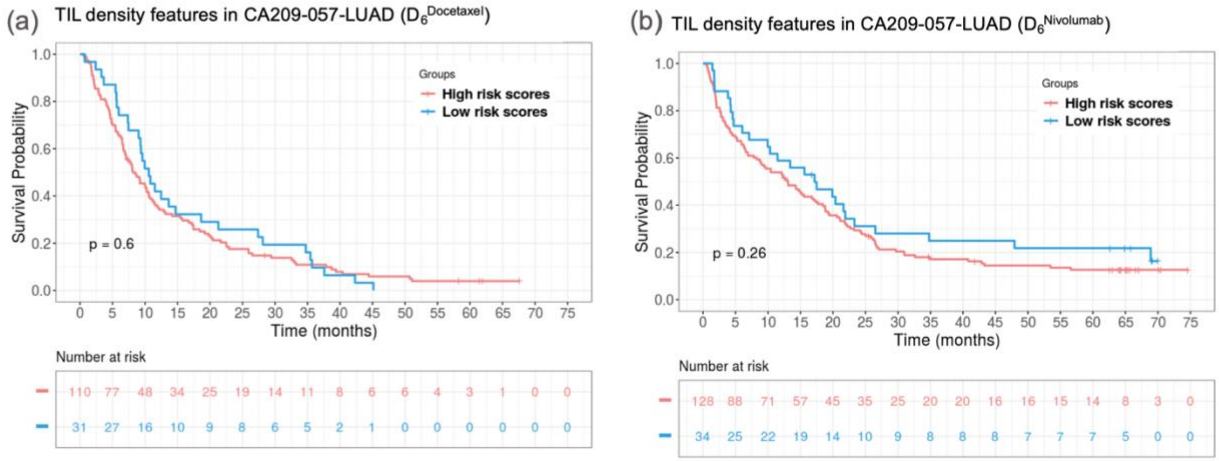

Supplementary Figure 8. Kaplan-Meier survival curves generated from the results of Cox model on the external validation set D_6. (a) represents the plot for 141 patients treated with Docetaxel (HR = 0.9 (95% CI = 0.6 – 1.34), C-index = 0.582 (95% CI 0.533 – 0.631, SE = 0.025) and (b) represents the plot for 162 patients treated with Nivolumab (HR = 0.78 (95% CI = 0.51 – 1.2), C-index = 0.547 (95% CI 0.495 – 0.6, SE = 0.027).

LUAD prognostic DenTIL features	Feature weight
Standard deviation of count of TILs in each grid when dividing the tile into a 5 x 5 grid (standard deviation across tiles)	-3.17
Maximum value of count of TILs in each grid when dividing the tile into a 5 x 5 grid (range across tiles)	-1.62
Intersected area between convex hull of TILs and non-TILs (range across tiles)	0.340
Minimum value of count of TILs in each grid when dividing the tile into a 5 x 5 grid (range across tiles)	0.286
The TIL density as reflected by the closeness of one TIL to its neighboring TILs (median across tiles)	0.216
Ratio of total area of TILs to total area of the tissue (range across tiles)	-0.122

LUSC prognostic graph/shape features	Feature weight
The entropy of the cell orientation of TIL (standard deviation across tiles)	0.351
Graph average of distance to three nearest neighbors of TILs (average across tiles)	0.335
Mean of Fourier shape descriptor features of TILs (average across tiles)	0.243
Standard Deviation of Fourier shape descriptor features of TILs (average across tiles)	0.127
Cell run length (local cell graphs decomposed into separate cliques comprising different number of connected cells) non-uniformity of TILs (range across tiles)	0.0222

LUSC prognostic SpaTIL features	Feature weight
Graph average of nearest neighbors of TILs within 20-pixel radius (average across tiles)	1.59
Among non-TIL clusters, the median number of five nearest neighboring TIL clusters (range across tiles)	0.722
Graph disorder of nearest neighbors of TILs within 50-pixel radius (standard deviation across tiles)	-0.558
Among TIL clusters, the median number of three nearest neighboring non-TIL clusters (median across tiles)	0.302
Median of the intersected area between TIL clusters and non-TIL clusters divided by non-TIL cluster areas (range across tiles)	-0.198
Minimum value of density of non-TIL clusters (range across tiles)	-0.167
Standard deviation of area of non-TIL clusters (average across tiles)	-0.118
The maximum ratio of the intersected area between TIL clusters and non-TIL clusters divided by the average area of TIL and non-TIL clusters (standard deviation across tiles)	-0.0802

Supplementary Table 1. The description of the most discriminant features from survival analysis experiments for TCGA dataset (D_1 and D_2). Each table was sorted by the absolute value of the feature weights. A positive weight implies a positive correlation with risk, and a negative weight implies a negative correlation with risk.

1	#Lymp/TissueArea
2	LympTotalArea/TissueArea
3	#Lymp/#TotalNuclei
4	MaxLympGroupingFactor
5	MinLympGroupingFactor
6	AvgLympGroupingFactor
7	StdLympGroupingFactor
8	MedianLympGroupingFactor
9	ModeLympGroupingFactor
10	NumHighlyGroupedLymp
11	#Lymp/TotalConvHullArea
12	LympConvHullArea/TotalConvHullArea
13	IntersectedAreaConvHullLymp&NonLymp
14	MaxDensityMatrixVal
15	MinDensityMatrixVal
16	AvgDensityMatrixVal
17	StdDensityMatrixVal
18	MedianDensityMatrixVal
19	ModeDensityMatrixVal

Supplementary Table 2. The complete list of all 19 DenTIL features. Note that “Lymp” represents lymphocytes, and “std” represents standard deviation. As an example of the naming convention, the first feature listed, “#Lymp/TissueArea”, means number of lymphocytes divided by the tissue area.

1	NumClusters_ lymp
2	NumClusters_ nonLymp
3	TotalAreaClusters_ lymp
4	MeanAreaClusters_ lymp
5	StdAreaClusters_ lymp
6	MedianAreaClusters_ lymp
7	MaxAreaClusters_ lymp
8	MinAreaClusters_ lymp
9	KurtosisAreaClusters_ lymp
10	SkewnessAreaClusters_ lymp
11	TotalDensityClusters_ lymp
12	MeanDensityClusters_ lymp
13	StdDensityClusters_ lymp
14	MedianDensityClusters_ lymp
15	MaxDensityClusters_ lymp
16	MinDensityClusters_ lymp
17	KurtosisDensityClusters_ lymp
18	SkewnessDensityClusters_ lymp
19	TotalAreaClusters_ nonLymp
20	MeanAreaClusters_ nonLymp
21	StdAreaClusters_ nonLymp
22	MedianAreaClusters_ nonLymp
23	MaxAreaClusters_ nonLymp
24	MinAreaClusters_ nonLymp
25	KurtosisAreaClusters_ nonLymp
26	SkewnessAreaClusters_ nonLymp
27	TotalDensityClusters_ nonLymp
28	MeanDensityClusters_ nonLymp
29	StdDensityClusters_ nonLymp
30	MedianDensityClusters_ nonLymp
31	MaxDensityClusters_ nonLymp
32	MinDensityClusters_ nonLymp
33	KurtosisDensityClusters_ nonLymp
34	SkewnessDensityClusters_ nonLymp
35	TotalIntersectedAreaClusters_ lymp & nonLymp
36	MeanIntersectedAreaClusters_ lymp & nonLymp
37	StdIntersectedAreaClusters_ lymp & nonLymp
38	MedianIntersectedAreaClusters_ lymp & nonLymp
39	MaxIntersectedAreaClusters_ lymp & nonLymp

40	MinIntersectedAreaClusters_ lymp & nonLymp
41	KurtosisIntersectedAreaClusters_ lymp & nonLymp
42	SkewnessIntersectedAreaClusters_ lymp & nonLymp
43	TotalRatioIntersectedAreaClusters_ lymp & nonLymp_ToArea_ lymp
44	MeanRatioIntersectedAreaClusters_ lymp & nonLymp_ToArea_ lymp
45	StdRatioIntersectedAreaClusters_ lymp & nonLymp_ToArea_ lymp
46	MedianRatioIntersectedAreaClusters_ lymp & nonLymp_ToArea_ lymp
47	MaxRatioIntersectedAreaClusters_ lymp & nonLymp_ToArea_ lymp
48	MinRatioIntersectedAreaClusters_ lymp & nonLymp_ToArea_ lymp
49	KurtosisRatioIntersectedAreaClusters_ lymp & nonLymp_ToArea_ lymp
50	SkewnessRatioIntersectedAreaClusters_ lymp & nonLymp_ToArea_ lymp
51	TotalRatioIntersectedAreaClusters_ lymp & nonLymp_ToArea_ nonLymp
52	MeanRatioIntersectedAreaClusters_ lymp & nonLymp_ToArea_ nonLymp
53	StdRatioIntersectedAreaClusters_ lymp & nonLymp_ToArea_ nonLymp
54	MedianRatioIntersectedAreaClusters_ lymp & nonLymp_ToArea_ nonLymp
55	MaxRatioIntersectedAreaClusters_ lymp & nonLymp_ToArea_ nonLymp
56	MinRatioIntersectedAreaClusters_ lymp & nonLymp_ToArea_ nonLymp
57	KurtosisRatioIntersectedAreaClusters_ lymp & nonLymp_ToArea_ nonLymp
58	SkewnessRatioIntersectedAreaClusters_ lymp & nonLymp_ToArea_ nonLymp
59	TotalRatioIntersectedAreaClusters_ lymp & nonLymp_ToAvgArea_ lymp & nonLymp
60	MeanRatioIntersectedAreaClusters_ lymp & nonLymp_ToAvgArea_ lymp & nonLymp
61	StdRatioIntersectedAreaClusters_ lymp & nonLymp_ToAvgArea_ lymp & nonLymp
62	MedianRatioIntersectedAreaClusters_ lymp & nonLymp_ToAvgArea_ lymp & nonLymp
63	MaxRatioIntersectedAreaClusters_ lymp & nonLymp_ToAvgArea_ lymp & nonLymp
64	MinRatioIntersectedAreaClusters_ lymp & nonLymp_ToAvgArea_ lymp & nonLymp
65	KurtosisRatioIntersectedAreaClusters_ lymp & nonLymp_ToAvgArea_ lymp & nonLymp
66	SkewnessRatioIntersectedAreaClusters_ lymp & nonLymp_ToAvgArea_ lymp & nonLymp
67	TotalPercentageClusters_ lymp_Surrounding_ lymp_Neighborhood1
68	TotalPercentageClusters_ nonLymp_Surrounding_ lymp_Neighborhood1
69	TotalPercentageClusters_ lymp_Surrounding_ lymp_Neighborhood2
70	TotalPercentageClusters_ nonLymp_Surrounding_ lymp_Neighborhood2
71	TotalPercentageClusters_ lymp_Surrounding_ lymp_Neighborhood3
72	TotalPercentageClusters_ nonLymp_Surrounding_ lymp_Neighborhood3
73	TotalPercentageClusters_ lymp_Surrounding_ lymp_Neighborhood4
74	TotalPercentageClusters_ nonLymp_Surrounding_ lymp_Neighborhood4

75	TotalPercentageClusters_ lymp_Surrounding_ lymp_Neighborhood5
76	TotalPercentageClusters_ nonLymp_Surrounding_ lymp_Neighborhood5
77	MeanPercentageClusters_ lymp_Surrounding_ lymp_Neighborhood1
78	MeanPercentageClusters_ nonLymp_Surrounding_ lymp_Neighborhood1
79	MeanPercentageClusters_ lymp_Surrounding_ lymp_Neighborhood2
80	MeanPercentageClusters_ nonLymp_Surrounding_ lymp_Neighborhood2
81	MeanPercentageClusters_ lymp_Surrounding_ lymp_Neighborhood3
82	MeanPercentageClusters_ nonLymp_Surrounding_ lymp_Neighborhood3
83	MeanPercentageClusters_ lymp_Surrounding_ lymp_Neighborhood4
84	MeanPercentageClusters_ nonLymp_Surrounding_ lymp_Neighborhood4
85	MeanPercentageClusters_ lymp_Surrounding_ lymp_Neighborhood5
86	MeanPercentageClusters_ nonLymp_Surrounding_ lymp_Neighborhood5
87	StdPercentageClusters_ lymp_Surrounding_ lymp_Neighborhood1
88	StdPercentageClusters_ nonLymp_Surrounding_ lymp_Neighborhood1
89	StdPercentageClusters_ lymp_Surrounding_ lymp_Neighborhood2
90	StdPercentageClusters_ nonLymp_Surrounding_ lymp_Neighborhood2
91	StdPercentageClusters_ lymp_Surrounding_ lymp_Neighborhood3
92	StdPercentageClusters_ nonLymp_Surrounding_ lymp_Neighborhood3
93	StdPercentageClusters_ lymp_Surrounding_ lymp_Neighborhood4
94	StdPercentageClusters_ nonLymp_Surrounding_ lymp_Neighborhood4
95	StdPercentageClusters_ lymp_Surrounding_ lymp_Neighborhood5
96	StdPercentageClusters_ nonLymp_Surrounding_ lymp_Neighborhood5
97	MedianPercentageClusters_ lymp_Surrounding_ lymp_Neighborhood1
98	MedianPercentageClusters_ nonLymp_Surrounding_ lymp_Neighborhood1
99	MedianPercentageClusters_ lymp_Surrounding_ lymp_Neighborhood2
100	MedianPercentageClusters_ nonLymp_Surrounding_ lymp_Neighborhood2
101	MedianPercentageClusters_ lymp_Surrounding_ lymp_Neighborhood3
102	MedianPercentageClusters_ nonLymp_Surrounding_ lymp_Neighborhood3
103	MedianPercentageClusters_ lymp_Surrounding_ lymp_Neighborhood4
104	MedianPercentageClusters_ nonLymp_Surrounding_ lymp_Neighborhood4
105	MedianPercentageClusters_ lymp_Surrounding_ lymp_Neighborhood5
106	MedianPercentageClusters_ nonLymp_Surrounding_ lymp_Neighborhood5
107	MaxPercentageClusters_ lymp_Surrounding_ lymp_Neighborhood1
108	MaxPercentageClusters_ nonLymp_Surrounding_ lymp_Neighborhood1
109	MaxPercentageClusters_ lymp_Surrounding_ lymp_Neighborhood2
110	MaxPercentageClusters_ nonLymp_Surrounding_ lymp_Neighborhood2
111	MaxPercentageClusters_ lymp_Surrounding_ lymp_Neighborhood3
112	MaxPercentageClusters_ nonLymp_Surrounding_ lymp_Neighborhood3
113	MaxPercentageClusters_ lymp_Surrounding_ lymp_Neighborhood4

114	MaxPercentageClusters_ nonLymp_Surrounding_ lymp_Neighborhood4
115	MaxPercentageClusters_ lymp_Surrounding_ lymp_Neighborhood5
116	MaxPercentageClusters_ nonLymp_Surrounding_ lymp_Neighborhood5
117	MinPercentageClusters_ lymp_Surrounding_ lymp_Neighborhood1
118	MinPercentageClusters_ nonLymp_Surrounding_ lymp_Neighborhood1
119	MinPercentageClusters_ lymp_Surrounding_ lymp_Neighborhood2
120	MinPercentageClusters_ nonLymp_Surrounding_ lymp_Neighborhood2
121	MinPercentageClusters_ lymp_Surrounding_ lymp_Neighborhood3
122	MinPercentageClusters_ nonLymp_Surrounding_ lymp_Neighborhood3
123	MinPercentageClusters_ lymp_Surrounding_ lymp_Neighborhood4
124	MinPercentageClusters_ nonLymp_Surrounding_ lymp_Neighborhood4
125	MinPercentageClusters_ lymp_Surrounding_ lymp_Neighborhood5
126	MinPercentageClusters_ nonLymp_Surrounding_ lymp_Neighborhood5
127	KurtosisPercentageClusters_ lymp_Surrounding_ lymp_Neighborhood1
128	KurtosisPercentageClusters_ nonLymp_Surrounding_ lymp_Neighborhood1
129	KurtosisPercentageClusters_ lymp_Surrounding_ lymp_Neighborhood2
130	KurtosisPercentageClusters_ nonLymp_Surrounding_ lymp_Neighborhood2
131	KurtosisPercentageClusters_ lymp_Surrounding_ lymp_Neighborhood3
132	KurtosisPercentageClusters_ nonLymp_Surrounding_ lymp_Neighborhood3
133	KurtosisPercentageClusters_ lymp_Surrounding_ lymp_Neighborhood4
134	KurtosisPercentageClusters_ nonLymp_Surrounding_ lymp_Neighborhood4
135	KurtosisPercentageClusters_ lymp_Surrounding_ lymp_Neighborhood5
136	KurtosisPercentageClusters_ nonLymp_Surrounding_ lymp_Neighborhood5
137	SkewnessPercentageClusters_ lymp_Surrounding_ lymp_Neighborhood1
138	SkewnessPercentageClusters_ nonLymp_Surrounding_ lymp_Neighborhood1
139	SkewnessPercentageClusters_ lymp_Surrounding_ lymp_Neighborhood2
140	SkewnessPercentageClusters_ nonLymp_Surrounding_ lymp_Neighborhood2
141	SkewnessPercentageClusters_ lymp_Surrounding_ lymp_Neighborhood3
142	SkewnessPercentageClusters_ nonLymp_Surrounding_ lymp_Neighborhood3
143	SkewnessPercentageClusters_ lymp_Surrounding_ lymp_Neighborhood4
144	SkewnessPercentageClusters_ nonLymp_Surrounding_ lymp_Neighborhood4
145	SkewnessPercentageClusters_ lymp_Surrounding_ lymp_Neighborhood5
146	SkewnessPercentageClusters_ nonLymp_Surrounding_ lymp_Neighborhood5
147	TotalPercentageClusters_ lymp_Surrounding_ nonLymp_Neighborhood1
148	TotalPercentageClusters_ nonLymp_Surrounding_ nonLymp_Neighborhood1
149	TotalPercentageClusters_ lymp_Surrounding_ nonLymp_Neighborhood2
150	TotalPercentageClusters_ nonLymp_Surrounding_ nonLymp_Neighborhood2
151	TotalPercentageClusters_ lymp_Surrounding_ nonLymp_Neighborhood3
152	TotalPercentageClusters_ nonLymp_Surrounding_ nonLymp_Neighborhood3

153	TotalPercentageClusters_ lymp_Surrounding_ nonLymp_Neighborhood4
154	TotalPercentageClusters_ nonLymp_Surrounding_ nonLymp_Neighborhood4
155	TotalPercentageClusters_ lymp_Surrounding_ nonLymp_Neighborhood5
156	TotalPercentageClusters_ nonLymp_Surrounding_ nonLymp_Neighborhood5
157	MeanPercentageClusters_ lymp_Surrounding_ nonLymp_Neighborhood1
158	MeanPercentageClusters_ nonLymp_Surrounding_ nonLymp_Neighborhood1
159	MeanPercentageClusters_ lymp_Surrounding_ nonLymp_Neighborhood2
160	MeanPercentageClusters_ nonLymp_Surrounding_ nonLymp_Neighborhood2
161	MeanPercentageClusters_ lymp_Surrounding_ nonLymp_Neighborhood3
162	MeanPercentageClusters_ nonLymp_Surrounding_ nonLymp_Neighborhood3
163	MeanPercentageClusters_ lymp_Surrounding_ nonLymp_Neighborhood4
164	MeanPercentageClusters_ nonLymp_Surrounding_ nonLymp_Neighborhood4
165	MeanPercentageClusters_ lymp_Surrounding_ nonLymp_Neighborhood5
166	MeanPercentageClusters_ nonLymp_Surrounding_ nonLymp_Neighborhood5
167	StdPercentageClusters_ lymp_Surrounding_ nonLymp_Neighborhood1
168	StdPercentageClusters_ nonLymp_Surrounding_ nonLymp_Neighborhood1
169	StdPercentageClusters_ lymp_Surrounding_ nonLymp_Neighborhood2
170	StdPercentageClusters_ nonLymp_Surrounding_ nonLymp_Neighborhood2
171	StdPercentageClusters_ lymp_Surrounding_ nonLymp_Neighborhood3
172	StdPercentageClusters_ nonLymp_Surrounding_ nonLymp_Neighborhood3
173	StdPercentageClusters_ lymp_Surrounding_ nonLymp_Neighborhood4
174	StdPercentageClusters_ nonLymp_Surrounding_ nonLymp_Neighborhood4
175	StdPercentageClusters_ lymp_Surrounding_ nonLymp_Neighborhood5
176	StdPercentageClusters_ nonLymp_Surrounding_ nonLymp_Neighborhood5
177	MedianPercentageClusters_ lymp_Surrounding_ nonLymp_Neighborhood1
178	MedianPercentageClusters_ nonLymp_Surrounding_ nonLymp_Neighborhood1
179	MedianPercentageClusters_ lymp_Surrounding_ nonLymp_Neighborhood2
180	MedianPercentageClusters_ nonLymp_Surrounding_ nonLymp_Neighborhood2
181	MedianPercentageClusters_ lymp_Surrounding_ nonLymp_Neighborhood3
182	MedianPercentageClusters_ nonLymp_Surrounding_ nonLymp_Neighborhood3
183	MedianPercentageClusters_ lymp_Surrounding_ nonLymp_Neighborhood4
184	MedianPercentageClusters_ nonLymp_Surrounding_ nonLymp_Neighborhood4
185	MedianPercentageClusters_ lymp_Surrounding_ nonLymp_Neighborhood5
186	MedianPercentageClusters_ nonLymp_Surrounding_ nonLymp_Neighborhood5
187	MaxPercentageClusters_ lymp_Surrounding_ nonLymp_Neighborhood1
188	MaxPercentageClusters_ nonLymp_Surrounding_ nonLymp_Neighborhood1
189	MaxPercentageClusters_ lymp_Surrounding_ nonLymp_Neighborhood2
190	MaxPercentageClusters_ nonLymp_Surrounding_ nonLymp_Neighborhood2
191	MaxPercentageClusters_ lymp_Surrounding_ nonLymp_Neighborhood3

192	MaxPercentageClusters_ nonLymp_Surrounding_ nonLymp_Neighborhood3
193	MaxPercentageClusters_ lymp_Surrounding_ nonLymp_Neighborhood4
194	MaxPercentageClusters_ nonLymp_Surrounding_ nonLymp_Neighborhood4
195	MaxPercentageClusters_ lymp_Surrounding_ nonLymp_Neighborhood5
196	MaxPercentageClusters_ nonLymp_Surrounding_ nonLymp_Neighborhood5
197	MinPercentageClusters_ lymp_Surrounding_ nonLymp_Neighborhood1
198	MinPercentageClusters_ nonLymp_Surrounding_ nonLymp_Neighborhood1
199	MinPercentageClusters_ lymp_Surrounding_ nonLymp_Neighborhood2
200	MinPercentageClusters_ nonLymp_Surrounding_ nonLymp_Neighborhood2
201	MinPercentageClusters_ lymp_Surrounding_ nonLymp_Neighborhood3
202	MinPercentageClusters_ nonLymp_Surrounding_ nonLymp_Neighborhood3
203	MinPercentageClusters_ lymp_Surrounding_ nonLymp_Neighborhood4
204	MinPercentageClusters_ nonLymp_Surrounding_ nonLymp_Neighborhood4
205	MinPercentageClusters_ lymp_Surrounding_ nonLymp_Neighborhood5
206	MinPercentageClusters_ nonLymp_Surrounding_ nonLymp_Neighborhood5
207	KurtosisPercentageClusters_ lymp_Surrounding_ nonLymp_Neighborhood1
208	KurtosisPercentageClusters_ nonLymp_Surrounding_ nonLymp_Neighborhood1
209	KurtosisPercentageClusters_ lymp_Surrounding_ nonLymp_Neighborhood2
210	KurtosisPercentageClusters_ nonLymp_Surrounding_ nonLymp_Neighborhood2
211	KurtosisPercentageClusters_ lymp_Surrounding_ nonLymp_Neighborhood3
212	KurtosisPercentageClusters_ nonLymp_Surrounding_ nonLymp_Neighborhood3
213	KurtosisPercentageClusters_ lymp_Surrounding_ nonLymp_Neighborhood4
214	KurtosisPercentageClusters_ nonLymp_Surrounding_ nonLymp_Neighborhood4
215	KurtosisPercentageClusters_ lymp_Surrounding_ nonLymp_Neighborhood5
216	KurtosisPercentageClusters_ nonLymp_Surrounding_ nonLymp_Neighborhood5
217	SkewnessPercentageClusters_ lymp_Surrounding_ nonLymp_Neighborhood1
218	SkewnessPercentageClusters_ nonLymp_Surrounding_ nonLymp_Neighborhood1
219	SkewnessPercentageClusters_ lymp_Surrounding_ nonLymp_Neighborhood2
220	SkewnessPercentageClusters_ nonLymp_Surrounding_ nonLymp_Neighborhood2
221	SkewnessPercentageClusters_ lymp_Surrounding_ nonLymp_Neighborhood3
222	SkewnessPercentageClusters_ nonLymp_Surrounding_ nonLymp_Neighborhood3
223	SkewnessPercentageClusters_ lymp_Surrounding_ nonLymp_Neighborhood4
224	SkewnessPercentageClusters_ nonLymp_Surrounding_ nonLymp_Neighborhood4
225	SkewnessPercentageClusters_ lymp_Surrounding_ nonLymp_Neighborhood5
226	SkewnessPercentageClusters_ nonLymp_Surrounding_ nonLymp_Neighborhood5
227	GraphAreaStandardDeviation_ lymp
228	GraphAreaAverage_ lymp

229	GraphAreaMinimum/Maximum_ lymp
230	GraphAreaDisorder_ lymp
231	GraphPerimeterStandardDeviation_ lymp
232	GraphPerimeterAverage_ lymp
233	GraphPerimeterMinimum/Maximum_ lymp
234	GraphPerimeterDisorder_ lymp
235	GraphChordStandardDeviation_ lymp
236	GraphChordAverage_ lymp
237	GraphChordMinimum/Maximum_ lymp
238	GraphChordDisorder_ lymp
239	GraphSideLengthMinimum/Maximum_ lymp
240	GraphSideLengthStandardDeviation_ lymp
241	GraphSideLengthAverage_ lymp
242	GraphSideLengthDisorder_ lymp
243	GraphTriangleAreaMinimum/Maximum_ lymp
244	GraphTriangleAreaStandardDeviation_ lymp
245	GraphTriangleAreaAverage_ lymp
246	GraphTriangleAreaDisorder_ lymp
247	GraphMSTEdgeLengthAverage_ lymp
248	GraphMSTEdgeLengthStandardDeviation_ lymp
249	GraphMSTEdgeLengthMinimum/Maximum_ lymp
250	GraphMSTEdgeLengthDisorder_ lymp
251	GraphAreaofpolygons_ lymp
252	GraphNumberofnuclei_ lymp
253	GraphDensityofNuclei_ lymp
254	GraphAveragedistanceto3NearestNeighbors_ lymp
255	GraphAveragedistanceto5NearestNeighbors_ lymp
256	GraphAveragedistanceto7NearestNeighbors_ lymp
257	GraphStandardDeviationdistanceto3NearestNeighbors_ lymp
258	GraphStandardDeviationdistanceto5NearestNeighbors_ lymp
259	GraphStandardDeviationdistanceto7NearestNeighbors_ lymp
260	GraphDisorderofdistanceto3NearestNeighbors_ lymp
261	GraphDisorderofdistanceto5NearestNeighbors_ lymp
262	GraphDisorderofdistanceto7NearestNeighbors_ lymp
263	GraphAvg.NearestNeighborsina10PixelRadius_ lymp
264	GraphAvg.NearestNeighborsina20PixelRadius_ lymp
265	GraphAvg.NearestNeighborsina30PixelRadius_ lymp
266	GraphAvg.NearestNeighborsina40PixelRadius_ lymp
267	GraphAvg.NearestNeighborsina50PixelRadius_ lymp

268	GraphStandardDeviationNearestNeighborsina10PixelRadius_ lymp
269	GraphStandardDeviationNearestNeighborsina20PixelRadius_ lymp
270	GraphStandardDeviationNearestNeighborsina30PixelRadius_ lymp
271	GraphStandardDeviationNearestNeighborsina40PixelRadius_ lymp
272	GraphStandardDeviationNearestNeighborsina50PixelRadius_ lymp
273	GraphDisorderofNearestNeighborsina10PixelRadius_ lymp
274	GraphDisorderofNearestNeighborsina20PixelRadius_ lymp
275	GraphDisorderofNearestNeighborsina30PixelRadius_ lymp
276	GraphDisorderofNearestNeighborsina40PixelRadius_ lymp
277	GraphDisorderofNearestNeighborsina50PixelRadius_ lymp
278	GraphAreaStandardDeviation_ nonLymp
279	GraphAreaAverage_ nonLymp
280	GraphAreaMinimum/Maximum_ nonLymp
281	GraphAreaDisorder_ nonLymp
282	GraphPerimeterStandardDeviation_ nonLymp
283	GraphPerimeterAverage_ nonLymp
284	GraphPerimeterMinimum/Maximum_ nonLymp
285	GraphPerimeterDisorder_ nonLymp
286	GraphChordStandardDeviation_ nonLymp
287	GraphChordAverage_ nonLymp
288	GraphChordMinimum/Maximum_ nonLymp
289	GraphChordDisorder_ nonLymp
290	GraphSideLengthMinimum/Maximum_ nonLymp
291	GraphSideLengthStandardDeviation_ nonLymp
292	GraphSideLengthAverage_ nonLymp
293	GraphSideLengthDisorder_ nonLymp
294	GraphTriangleAreaMinimum/Maximum_ nonLymp
295	GraphTriangleAreaStandardDeviation_ nonLymp
296	GraphTriangleAreaAverage_ nonLymp
297	GraphTriangleAreaDisorder_ nonLymp
298	GraphMSTEdgeLengthAverage_ nonLymp
299	GraphMSTEdgeLengthStandardDeviation_ nonLymp
300	GraphMSTEdgeLengthMinimum/Maximum_ nonLymp
301	GraphMSTEdgeLengthDisorder_ nonLymp
302	GraphAreaofpolygons_ nonLymp
303	GraphNumberofnuclei_ nonLymp
304	GraphDensityofNuclei_ nonLymp
305	GraphAveragedistanceto3NearestNeighbors_ nonLymp
306	GraphAveragedistanceto5NearestNeighbors_ nonLymp

307	GraphAveragedistanceto7NearestNeighbors_ nonLymp
308	GraphStandardDeviationdistanceto3NearestNeighbors_ nonLymp
309	GraphStandardDeviationdistanceto5NearestNeighbors_ nonLymp
310	GraphStandardDeviationdistanceto7NearestNeighbors_ nonLymp
311	GraphDisorderofdistanceto3NearestNeighbors_ nonLymp
312	GraphDisorderofdistanceto5NearestNeighbors_ nonLymp
313	GraphDisorderofdistanceto7NearestNeighbors_ nonLymp
314	GraphAvg.NearestNeighborsina10PixelRadius_ nonLymp
315	GraphAvg.NearestNeighborsina20PixelRadius_ nonLymp
316	GraphAvg.NearestNeighborsina30PixelRadius_ nonLymp
317	GraphAvg.NearestNeighborsina40PixelRadius_ nonLymp
318	GraphAvg.NearestNeighborsina50PixelRadius_ nonLymp
319	GraphStandardDeviationNearestNeighborsina10PixelRadius_ nonLymp
320	GraphStandardDeviationNearestNeighborsina20PixelRadius_ nonLymp
321	GraphStandardDeviationNearestNeighborsina30PixelRadius_ nonLymp
322	GraphStandardDeviationNearestNeighborsina40PixelRadius_ nonLymp
323	GraphStandardDeviationNearestNeighborsina50PixelRadius_ nonLymp
324	GraphDisorderofNearestNeighborsina10PixelRadius_ nonLymp
325	GraphDisorderofNearestNeighborsina20PixelRadius_ nonLymp
326	GraphDisorderofNearestNeighborsina30PixelRadius_ nonLymp
327	GraphDisorderofNearestNeighborsina40PixelRadius_ nonLymp
328	GraphDisorderofNearestNeighborsina50PixelRadius_ nonLymp
329	IntersectionArea_ lymp & nonLymp
330	RatioIntersectedArea_ lymp & nonLymp_ToArea_ lymp
331	RatioIntersectedArea_ lymp & nonLymp_ToArea_ nonLymp
332	RatioIntersectedArea_ lymp & nonLymp_ToAvgArea_ lymp & nonLymp
333	NumCentroidsClusters_ lymp_InConvHull_ nonLymp
334	NumCentroidsClusters_ nonLymp_InConvHull_ lymp
335	TotalGroupingFactor_ lymp
336	MeanGroupingFactor_ lymp
337	StdGroupingFactor_ lymp
338	MedianGroupingFactor_ lymp
339	MaxGroupingFactor_ lymp
340	MinGroupingFactor_ lymp
341	KurtosisGroupingFactor_ lymp
342	SkewnessGroupingFactor_ lymp
343	TotalGroupingFactor_ nonLymp
344	MeanGroupingFactor_ nonLymp
345	StdGroupingFactor_ nonLymp

346	MedianGroupingFactor_ nonLymp
347	MaxGroupingFactor_ nonLymp
348	MinGroupingFactor_ nonLymp
349	KurtosisGroupingFactor_ nonLymp
350	SkewnessGroupingFactor_ nonLymp

Supplementary Table 3. The complete list of all 350 SpaTIL features. Note that "lymp" represents lymphocytes, and "std" represents standard deviation. As an example of the naming convention, the fifth feature listed, "StdAreaClusters_lymp", means the standard deviation of area of lymphocyte clusters.

1	Voronoi:Area Standard Deviation
2	Voronoi:Area Average
3	Voronoi:Area Minimum / Maximum
4	Voronoi:Area Disorder
5	Voronoi:Perimeter Standard Deviation
6	Voronoi:Perimeter Average
7	Voronoi:Perimeter Minimum / Maximum
8	Voronoi:Perimeter Disorder
9	Voronoi:Chord Standard Deviation
10	Voronoi:Chord Average
11	Voronoi:Chord Minimum / Maximum
12	Voronoi:Chord Disorder
13	Delaunay:Side Length Minimum / Maximum
14	Delaunay:Side Length Standard Deviation
15	Delaunay:Side Length Average
16	Delaunay:Side Length Disorder
17	Delaunay:Triangle Area Minimum / Maximum
18	Delaunay:Triangle Area Standard Deviation
19	Delaunay:Triangle Area Average
20	Delaunay:Triangle Area Disorder
21	MST:MST Edge Length Average
22	MST:MST Edge Length Standard Deviation
23	MST:MST Edge Length Minimum / Maximum
24	MST:MST Edge Length Disorder
25	Arch:Area of polygons
26	Arch:Number of Polygons
27	Arch:Density of Polygons
28	Arch:Average distance to 3 Nearest Neighbors
29	Arch:Average distance to 5 Nearest Neighbors
30	Arch:Average distance to 7 Nearest Neighbors
31	Arch:Standard Deviation distance to 3 Nearest Neighbors
32	Arch:Standard Deviation distance to 5 Nearest Neighbors
33	Arch:Standard Deviation distance to 7 Nearest Neighbors
34	Arch:Disorder of distance to 3 Nearest Neighbors
35	Arch:Disorder of distance to 5 Nearest Neighbors
36	Arch:Disorder of distance to 7 Nearest Neighbors
37	Arch:Avg. Nearest Neighbors in a 10 Pixel Radius
38	Arch:Avg. Nearest Neighbors in a 20 Pixel Radius
39	Arch:Avg. Nearest Neighbors in a 30 Pixel Radius

40	Arch:Avg. Nearest Neighbors in a 40 Pixel Radius
41	Arch:Avg. Nearest Neighbors in a 50 Pixel Radius
42	Arch:Standard Deviation Nearest Neighbors in a 10 Pixel Radius
43	Arch:Standard Deviation Nearest Neighbors in a 20 Pixel Radius
44	Arch:Standard Deviation Nearest Neighbors in a 30 Pixel Radius
45	Arch:Standard Deviation Nearest Neighbors in a 40 Pixel Radius
46	Arch:Standard Deviation Nearest Neighbors in a 50 Pixel Radius
47	Arch:Disorder of Nearest Neighbors in a 10 Pixel Radius
48	Arch:Disorder of Nearest Neighbors in a 20 Pixel Radius
49	Arch:Disorder of Nearest Neighbors in a 30 Pixel Radius
50	Arch:Disorder of Nearest Neighbors in a 40 Pixel Radius
51	Arch:Disorder of Nearest Neighbors in a 50 Pixel Radius
52	Morph:Mean Area Ratio
53	Morph:Mean Distance Ratio
54	Morph:Mean Standard Deviation of Distance
55	Morph:Mean Variance of Distance
56	Morph:Mean Long/Short Distance Ratio
57	Morph:Mean Perimeter Ratio
58	Morph:Mean Smoothness
59	Morph:Mean Invariant Moment 1
60	Morph:Mean Invariant Moment 2
61	Morph:Mean Invariant Moment 3
62	Morph:Mean Invariant Moment 4
63	Morph:Mean Invariant Moment 5
64	Morph:Mean Invariant Moment 6
65	Morph:Mean Invariant Moment 7
66	Morph:Mean Fractal Dimension
67	Morph:Mean Fourier Descriptor 1
68	Morph:Mean Fourier Descriptor 2
69	Morph:Mean Fourier Descriptor 3
70	Morph:Mean Fourier Descriptor 4
71	Morph:Mean Fourier Descriptor 5
72	Morph:Mean Fourier Descriptor 6
73	Morph:Mean Fourier Descriptor 7
74	Morph:Mean Fourier Descriptor 8
75	Morph:Mean Fourier Descriptor 9
76	Morph:Mean Fourier Descriptor 10
77	Morph:Standard Deviation Area Ratio
78	Morph:Standard Deviation Distance Ratio

79	Morph:Standard Deviation Standard Deviation of Distance
80	Morph:Standard Deviation Variance of Distance
81	Morph:Standard Deviation Long/Short Distance Ratio
82	Morph:Standard Deviation Perimeter Ratio
83	Morph:Standard Deviation Smoothness
84	Morph:Standard Deviation Invariant Moment 1
85	Morph:Standard Deviation Invariant Moment 2
86	Morph:Standard Deviation Invariant Moment 3
87	Morph:Standard Deviation Invariant Moment 4
88	Morph:Standard Deviation Invariant Moment 5
89	Morph:Standard Deviation Invariant Moment 6
90	Morph:Standard Deviation Invariant Moment 7
91	Morph:Standard Deviation Fractal Dimension
92	Morph:Standard Deviation Fourier Descriptor 1
93	Morph:Standard Deviation Fourier Descriptor 2
94	Morph:Standard Deviation Fourier Descriptor 3
95	Morph:Standard Deviation Fourier Descriptor 4
96	Morph:Standard Deviation Fourier Descriptor 5
97	Morph:Standard Deviation Fourier Descriptor 6
98	Morph:Standard Deviation Fourier Descriptor 7
99	Morph:Standard Deviation Fourier Descriptor 8
100	Morph:Standard Deviation Fourier Descriptor 9
101	Morph:Standard Deviation Fourier Descriptor 10
102	Morph:Median Area Ratio
103	Morph:Median Distance Ratio
104	Morph:Median Standard Deviation of Distance
105	Morph:Median Variance of Distance
106	Morph:Median Long/Short Distance Ratio
107	Morph:Median Perimeter Ratio
108	Morph:Median Smoothness
109	Morph:Median Invariant Moment 1
110	Morph:Median Invariant Moment 2
111	Morph:Median Invariant Moment 3
112	Morph:Median Invariant Moment 4
113	Morph:Median Invariant Moment 5
114	Morph:Median Invariant Moment 6
115	Morph:Median Invariant Moment 7
116	Morph:Median Fractal Dimension
117	Morph:Median Fourier Descriptor 1

118	Morph:Median Fourier Descriptor 2
119	Morph:Median Fourier Descriptor 3
120	Morph:Median Fourier Descriptor 4
121	Morph:Median Fourier Descriptor 5
122	Morph:Median Fourier Descriptor 6
123	Morph:Median Fourier Descriptor 7
124	Morph:Median Fourier Descriptor 8
125	Morph:Median Fourier Descriptor 9
126	Morph:Median Fourier Descriptor 10
127	Morph:Min / Max Area Ratio
128	Morph:Min / Max Distance Ratio
129	Morph:Min / Max Standard Deviation of Distance
130	Morph:Min / Max Variance of Distance
131	Morph:Min / Max Long/Short Distance Ratio
132	Morph:Min / Max Perimeter Ratio
133	Morph:Min / Max Smoothness
134	Morph:Min / Max Invariant Moment 1
135	Morph:Min / Max Invariant Moment 2
136	Morph:Min / Max Invariant Moment 3
137	Morph:Min / Max Invariant Moment 4
138	Morph:Min / Max Invariant Moment 5
139	Morph:Min / Max Invariant Moment 6
140	Morph:Min / Max Invariant Moment 7
141	Morph:Min / Max Fractal Dimension
142	Morph:Min / Max Fourier Descriptor 1
143	Morph:Min / Max Fourier Descriptor 2
144	Morph:Min / Max Fourier Descriptor 3
145	Morph:Min / Max Fourier Descriptor 4
146	Morph:Min / Max Fourier Descriptor 5
147	Morph:Min / Max Fourier Descriptor 6
148	Morph:Min / Max Fourier Descriptor 7
149	Morph:Min / Max Fourier Descriptor 8
150	Morph:Min / Max Fourier Descriptor 9
151	Morph:Min / Max Fourier Descriptor 10
152	CGT:mean tensor contrast_energy
153	CGT:standard deviation tensor contrast_energy
154	CGT:range tensor contrast_energy
155	CGT:mean tensor contrast_inverse_moment
156	CGT:standard deviation tensor contrast_inverse_moment

157	CGT:range tensor contrast_inverse_moment
158	CGT:mean tensor contrast_ave
159	CGT:standard deviation tensor contrast_ave
160	CGT:range tensor contrast_ave
161	CGT:mean tensor contrast_var
162	CGT:standard deviation tensor contrast_var
163	CGT:range tensor contrast_var
164	CGT:mean tensor contrast_entropy
165	CGT:standard deviation tensor contrast_entropy
166	CGT:range tensor contrast_entropy
167	CGT:mean tensor intensity_ave
168	CGT:standard deviation tensor intensity_ave
169	CGT:range tensor intensity_ave
170	CGT:mean tensor intensity_variance
171	CGT:standard deviation tensor intensity_variance
172	CGT:range tensor intensity_variance
173	CGT:mean tensor intensity_entropy
174	CGT:standard deviation tensor intensity_entropy
175	CGT:range tensor intensity_entropy
176	CGT:mean tensor entropy,
177	CGT:standard deviation tensor entropy,
178	CGT:range tensor entropy,
179	CGT:mean tensor energy
180	CGT:standard deviation tensor energy
181	CGT:range tensor energy
182	CGT:mean tensor correlation
183	CGT:standard deviation tensor correlation
184	CGT:range tensor correlation
185	CGT:mean tensor information_measure1
186	CGT:standard deviation tensor information_measure1
187	CGT:range tensor information_measure1
188	CGT:mean tensor information_measure2
189	CGT:standard deviation tensor information_measure2
190	CGT:range tensor information_measure2
191	GSG:Number of Nodes
192	GSG:Number of Edges
193	GSG:Average Degree
194	GSG:Average Eccentricity
195	GSG:Diameter

196	GSG:Radius
197	GSG:Average Eccentricity 90\%
198	GSG:Diameter 90\%
199	GSG:Radius 90\%
200	GSG:Average Path Length
201	GSG:Clustering Coefficient C
202	GSG:Clustering Coefficient D
203	GSG:Clustering Coefficient E
204	GSG:Number of connected components
205	GSG:giant connected component ratio
206	GSG:average connected component size
207	GSG:number isolated nodes
208	GSG:percentage isolated nodes
209	GSG:number end nodes
210	GSG:percentage end nodes
211	GSG:number central nodes
212	GSG:percentage central nodes
213	GSG:mean edge length
214	GSG:standard deviation edge length
215	GSG:skewness edge length
216	GSG:kurtosis edge length
217	CRL-mean(Short Run Emphasis)
218	CRL-mean(Long Run Emphasis)
219	CRL-mean(Run Length Nonuniformity)
220	CRL-mean(Run Percentage)
221	CRL-median(Short Run Emphasis)
222	CRL-median(Long Run Emphasis)
223	CRL-median(Run Length Nonuniformity)
224	CRL-median(Run Percentage)
225	CRL-std(Short Run Emphasis)
226	CRL-std(Long Run Emphasis)
227	CRL-std(Run Length Nonuniformity)
228	CRL-std(Run Percentage)
229	CRL-range(Short Run Emphasis)
230	CRL-range(Long Run Emphasis)
231	CRL-range(Run Length Nonuniformity)
232	CRL-range(Run Percentage)
233	CRL-kurtosis(Short Run Emphasis)
234	CRL-kurtosis(Long Run Emphasis)

235	CRL-kurtosis(Run Length Nonuniformity)
236	CRL-kurtosis(Run Percentage)
237	CRL-skewness(Short Run Emphasis)
238	CRL-skewness(Long Run Emphasis)
239	CRL-skewness(Run Length Nonuniformity)
240	CRL-skewness(Run Percentage)

Supplementary Table 4. The complete list of all 240 graph- and shape-based features. 1-51 are global graph-based features, 52-151 are shape-based features, 152-190 are cell orientation entropy features, 191-216 are cell cluster graph features, 217-240 are cell run length features.
