# Supplementary material for: Image analysis reveals molecularly distinct patterns of TILs in NSCLC associated with treatment outcome
Source: NPJ Precis Oncol. 2022 Jun 3;6:33. doi: 10.1038/s41698-022-00277-5 (PMC9166700; doi:10.1038/s41698-022-00277-5)
Supplement: Supplementary file 2 — REPORTING SUMMARY [file 41698_2022_277_MOESM2_ESM.pdf]

nature portfolio

Corresponding author(s): DBPR NPJPRECISIONONCOLOGY-01505R

Last updated by author(s): Feb 16, 2022

Reporting Summary

Nature Portfolio wishes to improve the reproducibility of the work that we publish. This form provides structure for consistency and transparency in reporting. For further information on Nature Portfolio policies, see our Editorial Policies and the Editorial Policy Checklist.

Statistics

For all statistical analyses, confirm that the following items are present in the figure legend, table legend, main text, or Methods section.

n/a	Confirmed	
☐	☒	The exact sample size (n) for each experimental group/condition, given as a discrete number and unit of measurement
☐	☒	A statement on whether measurements were taken from distinct samples or whether the same sample was measured repeatedly
☐	☒	The statistical test(s) used AND whether they are one- or two-sided<br>*Only common tests should be described solely by name; describe more complex techniques in the Methods section.*
☒	☐	A description of all covariates tested
☐	☒	A description of any assumptions or corrections, such as tests of normality and adjustment for multiple comparisons
☐	☒	A full description of the statistical parameters including central tendency (e.g. means) or other basic estimates (e.g. regression coefficient) AND variation (e.g. standard deviation) or associated estimates of uncertainty (e.g. confidence intervals)
☐	☒	For null hypothesis testing, the test statistic (e.g. F, t, r) with confidence intervals, effect sizes, degrees of freedom and P value noted<br>*Give P values as exact values whenever suitable.*
☒	☐	For Bayesian analysis, information on the choice of priors and Markov chain Monte Carlo settings
☐	☒	For hierarchical and complex designs, identification of the appropriate level for tests and full reporting of outcomes
☐	☒	Estimates of effect sizes (e.g. Cohen's d, Pearson's r), indicating how they were calculated

Our web collection on statistics for biologists contains articles on many of the points above.

Software and code

Policy information about availability of computer code

Data collection	RTCGAToolbox (Bioconductor 3.12) was used to collect the TCGA data
Data analysis	MATLAB2020b, Python3.7, R4.0.0

For manuscripts utilizing custom algorithms or software that are central to the research but not yet described in published literature, software must be made available to editors and reviewers. We strongly encourage code deposition in a community repository (e.g. GitHub). See the Nature Portfolio guidelines for submitting code & software for further information.

Data

Policy information about availability of data

All manuscripts must include a data availability statement. This statement should provide the following information, where applicable:

- Accession codes, unique identifiers, or web links for publicly available datasets
- A description of any restrictions on data availability
- For clinical datasets or third party data, please ensure that the statement adheres to our policy

D1 and D2 were generated by TCGA Research Network (http://cancergenome.nih.gov/), and they have made them publicly available. Since the cases from the involved institutions are protected through institutional compliance, the clinical repository of cases can only be shared per specific institutional review board (IRB) requirements. Upon reasonable request, a data sharing agreement can be initiated between the interested parties and the clinical institution following institution-specific guidelines.

Field-specific reporting

Please select the one below that is the best fit for your research. If you are not sure, read the appropriate sections before making your selection.

☒ Life sciences ☐ Behavioural & social sciences ☐ Ecological, evolutionary & environmental sciences

For a reference copy of the document with all sections, see nature.com/documents/nr-reporting-summary-flat.pdf

Life sciences study design

All studies must disclose on these points even when the disclosure is negative.

Sample size	Six datasets were used for this study, including 421 TCGA-LUAD (D1), 438 TCGA-LUSC (D2), 62 Yale-LUAD (D3), 21 Yale-LUSC (D4) cases, 100 UBern-LUAD (D5), and 303 CA209-057 (D6) cases suitable for the downstream analysis.
Data exclusions	For D1 and D2, inclusion criteria comprised availability of immune scores and overall survival information. For D3, D4, and D5, the inclusion criterion is the availability of overall survival information. For D6, the inclusion criteria are the availability of overall survival information, response status and histologic subtype being LUAD.
Replication	We independently validated our prognostic model from training set on the independent test set to evaluate the robustness of the built model.
Randomization	Our study doesn't involve random allocation of patient samples into different experimental groups. The covariate was controlled by checking the consistency in the prognostic value of morphologic patterns found across early and late stage disease in both LUAD and LUSC.
Blinding	During the image analysis and model construction process, we were blinded to the outcome data for the independent test set.

Reporting for specific materials, systems and methods

We require information from authors about some types of materials, experimental systems and methods used in many studies. Here, indicate whether each material, system or method listed is relevant to your study. If you are not sure if a list item applies to your research, read the appropriate section before selecting a response.

Materials & experimental systems

n/a	Involved in the study	
☒	☐	Antibodies
☒	☐	Eukaryotic cell lines
☒	☐	Palaeontology and archaeology
☒	☐	Animals and other organisms
☐	☒	Human research participants
☒	☐	Clinical data
☒	☐	Dual use research of concern

Methods

n/a	Involved in the study	
☒	☐	ChIP-seq
☒	☐	Flow cytometry
☒	☐	MRI-based neuroimaging

Human research participants

Policy information about studies involving human research participants

Population characteristics	The TCGA and Yale cohorts consist of patients diagnosed with mostly early-stage lung adenocarcinomas or squamous cell carcinomas; The UBern cohort consists of late-stage lung adenocarcinomas. A subset of 63 patients was treated with chemotherapy prior resection of which 57 patients in neoadjuvant intention and 60 patients were primary resected LUAD with pathologically confirmed infiltration of lymph nodes of at least the mediastinal level (indication of a locally-advanced stage). Patients of the neoadjuvant subset received platinum-based chemotherapy in different combinations: 1) Cisplatin plus Docetaxel, 2) Carboplatin plus Paclitaxel, 3) Cisplatin plus Pemetrexed, 4) Cisplatin plus Gemcitabine, 5) Cisplatin plus Vinorelbine, 6) Cisplatin plus Etoposide, and 7) other. The CA209-057 cohort used in this study is a phase 3 randomized clinical trial consisting of late-stage lung adenocarcinomas treated with either Nivolumab (n = 162) or Docetaxel (n = 141) after failure of previous platinum-based chemotherapy.
Recruitment	For the TCGA data, see https://www.cancer.gov/about-nci/organization/ccg/research/structural-genomics/tcga/history/policies/tcga-human-subjects-data-policies.pdf under Background section. For the Yale data, non-small cell lung cancer (NSCLC) participants were retrospectively recruited at Yale Pathology between 1988 and 2003. For the Bern cohort, consecutive patients diagnosed with NSCLC at the Institute of Pathology Bern between 2000 and 2016 and either receiving neoadjuvant chemo(radio)therapy or having a locally-advanced tumor stage (defined by pN2 or higher) were retrospectively included. For CA209-057, recruited patients are those who had advanced non-squamous NSCLC treated with either Nivolumab or Docetaxel after failure of previous platinum-based chemotherapy.

Ethics oversight	Corresponding clinicopathologic and outcome information from patients was obtained from the institutions at which the datasets were collected after obtaining the respective institutional review board approvals.

Note that full information on the approval of the study protocol must also be provided in the manuscript.
